# 8-Modified-2′-Deoxyadenosine Analogues Induce Delayed Polymerization Arrest during HIV-1 Reverse Transcription

**DOI:** 10.1371/journal.pone.0027456

**Published:** 2011-11-07

**Authors:** Valérie Vivet-Boudou, Catherine Isel, Marwan Sleiman, Redmond Smyth, Nouha Ben Gaied, Patrick Barhoum, Géraldine Laumond, Guillaume Bec, Matthias Götte, Johnson Mak, Anne-Marie Aubertin, Alain Burger, Roland Marquet

**Affiliations:** 1 Architecture et Réactivité de l'ARN, Institut de Biologie Moléculaire et Cellulaire, Université de Strasbourg, Centre National de la Recherche Scientifique, Strasbourg, France; 2 Centre for Virology, Burnet Institute, Melbourne, Australia; 3 Laboratoire de Chimie des Molécules Bioactives et des Arômes, Institut de Chimie de Nice, Université de Nice Sophia Antipolis, Centre National de la Recherche Scientifique, Nice, France; 4 Laboratoire de Virologie, Faculté de Médecine, Université de Strasbourg, Institut National de la Santé et de la Recherche Médicale, Strasbourg, France; 5 Department of Biochemistry, McGill University, Montreal, Canada; Institut Pasteur, France

## Abstract

The occurrence of resistant viruses to any of the anti-HIV-1 compounds used in the current therapies against AIDS underlies the urge for the development of new drug targets and/or new drugs acting through novel mechanisms. While all anti-HIV-1 nucleoside analogues in clinical use and in clinical trials rely on ribose modifications for activity, we designed nucleosides with a natural deoxyribose moiety and modifications of position 8 of the adenine base. Such modifications might induce a steric clash with helix αH in the thumb domain of the p66 subunit of HIV-1 RT at a distance from the catalytic site, causing delayed chain termination. Eleven new 2′-deoxyadenosine analogues modified on position 8 of the purine base were synthesized and tested *in vitro* and in cell-based assays. In this paper we demonstrate for the first time that chemical modifications on position 8 of 2′-deoxyadenosine induce delayed chain termination *in vitro,* and also inhibit DNA synthesis when incorporated in a DNA template strand. Furthermore, one of them had moderate anti-HIV-1 activity in cell-culture. Our results constitute a proof of concept indicating that modification on the base moiety of nucleosides can induce delayed polymerization arrest and inhibit HIV-1 replication.

## Introduction

Reverse transcription of the single-stranded genomic RNA into double-stranded DNA that will be integrated into the host genome is a key step of HIV-1 replication. This process is performed by the virally encoded reverse transcriptase (RT), which possesses RNA- and DNA-dependant DNA polymerase activity as well as RNase H activity [1].

After three decades of research, Highly Active Anti-Retroviral Therapy (HAART) [2], [3] is the best option to treat HIV-1-infected individuals. This therapeutic strategy combines three to five compounds mostly targeting RT and the viral protease. HAART quickly and strongly reduces the viral load, but it does not eradicate HIV-1 and drug-therapy is life-long [4]. Despite the existence of new drugs targeting viral entry (enfuvirtide [5] and maraviroc [6]) and integration (raltegravir [7]), that are generally used in later stages during treatment or for patients who failed the standard therapies, RT remains a major target of antiviral agents, with presently 12 clinically approved drugs. These drugs are divided into the two broad classes of nucleoside (and nucleotide) reverse transcriptase inhibitors (NRTIs) and non-nucleoside reverse transcriptase inhibitors (NNRTIs). NNRTIs are allosteric non-competitive RT inhibitors [8], [9] whereas all the approved NRTIs are competitive analogues of the natural dNTP substrates of RT. Once incorporated into the elongating DNA chain, they act as chain terminators, due to the lack of a 3′-OH group (for reviews, see [10], [11], [12]).

HIV-1 actively replicates in untreated infected individuals, and RT, which lacks proof-reading activity, is a highly error prone enzyme [13], [14]. As a consequence, suboptimal therapies lead to the emergence of resistant viruses, and a significant proportion of individuals are *primo*-infected with drug-resistant HIV-1 strains. In the case of NRTIs, resistance mechanisms fall into two classes (for reviews, see [10], [12], [15], [16]): (1) *decreased incorporation efficiency of the triphosphorylated form of the nucleoside analogue*, by enhanced discrimination of the NRTI with comparison to the natural dNTPs, either due to decreased binding of the NRTI and/or to a diminished rate of incorporation; (2) *removal of the NRTI from the end of the newly synthesized DNA* by RT, due to phosphorolysis, the reverse reaction of polymerization. Excision of 3′-azido-3′-deoxythymidine (AZT), and to a lesser extend 2′,3′-didehydro-2′,3′-dideoxythymidine (d4T), from the 3′ end of an elongating DNA primer is facilitated by a set of up to six mutations in the *pol* gene, including M41L, D67N, K70R, L210W, T215F/Y and K219E/Q, collectively referred to as Thymidine-analogue resistant mutations (TAMs). It has been shown that AZT-resistant RT is not only able to use PPi, but also a nucleotide triphosphate as PPi donor, most likely ATP *in vivo*, to excise the chain terminator (for a review, see [17]).

The inevitable occurrence of resistance to NRTIs, and indeed to all anti-HIV-1 drugs, underlies the need for new drugs directed against different targets and/or acting against existing targets *via* alternative mechanisms of action. In the case of HIV-1 RT, several NNRTIs are in late stage clinical trials and the RNase H activity of the enzyme is also considered to be an attractive target for the development of antiretrovirals (for recent reviews, see [10], [11], [12]).

One important consideration for the development of new inhibitors is their efficacy against existing drug-resistant RTs. Nucleoside analogues that (1) are incorporated into DNA by RT, (2) do not block DNA synthesis at their point of incorporation, and (3) block DNA synthesis only when a few natural dNTPs have been added after them should, to a large extent, be efficient against resistant RT acting through the excision mechanism. Such compounds have been developed recently and are called “delayed chain terminators” or DCTs. The first compound to display delayed polymerization arrest activity *in vitro* was a fixed conformation 2′-deoxyadenosine analogue, where the pseudosugar ring is locked in the North conformation [18], [19]. The triphosphorylated form of this nucleoside analogue is efficiently used as a substrate by HIV-1 RT *in vitro* and induces partial polymerization arrest when 2 or 3 more nucleotides are incorporated thereafter. It is also relatively resistant to excision by wild-type (WT) and resistant RTs. However this compound is not a drug candidate since it is poorly phosphorylated in cultured cells. Besides, several groups have investigated the properties of NRTIs bearing 4′ modifications on the pseudosugar ring. First [20] and second generation 4′-ethynyl-substituted compounds [21] displayed promising antiviral activities [22] and low cytotoxic effects [23]. Recently, it was shown that 4′-ethynyl-2-fluoro-2′-deoxyadenosine triphosphate inhibits RT translocation [24]. 4′-Methyl and 4′-ethyl-thymidine and adenosine also inhibit HIV-1 RT *in vitro* and *in vivo*, but in a temporal rather than a spatial sense and are therefore not strict delayed chain terminators [25], [26]. Finally, the guanosine analogue entecavir (ETV), a potent antiviral used to treat hepatitis B virus infected patients, which retains its 3′-OH group, has recently been shown to inhibit HIV-1 RT [27]. Interestingly, chain termination three nucleotides after the ETV incorporation site is the major mechanism of inhibition, *in vitro* [27], most likely because the 3′-end of the primer is being “repelled” from the active site. In addition, the presence of ETV at a distance from the catalytic site protects the elongating primer from excision by TAM-containing RTs, making it the first inhibitor acting by delayed chain termination that is active against HIV-1 in cell culture and in the clinic [27], [28].

The NRTIs used in the clinic and all DCTs developed so far are modified on their sugar moiety, keeping open the possibility of HIV-1 RT might develop cross-resistance to classical NRTIs and DCTs: for instance both 2′,3′-dideoxy-3′-thiacytidine (3TC) and ETV select the M184V resistance mutation in HIV-1 RT [29]. Here we tested the possibility of developing DCTs with a natural deoxyribose moiety and a modified base. We reasoned that such compounds might induce delayed chain termination because of a steric clash with helix αH in the thumb domain of the p66 subunit of HIV-1 RT and early mutational analysis of helix αH has indeed proven its importance for polymerization [30]. Several groups have investigated the synthesis of 8-substituted-2′-dA derivatives [31], [32], [33] but few antiviral data have been reported so far for these compounds. We designed, synthesized, and tested eleven of these analogues of 2′-deoxyadenosine. Most of them induce delayed chain termination *in vitro*, and one has a moderate anti HIV-1 activity in cell culture. Our results constitute a proof of concept indicating that modification on the base moiety of nucleosides can induce delayed polymerization arrest and inhibit HIV-1 replication.

## Results

**Figure 1:**
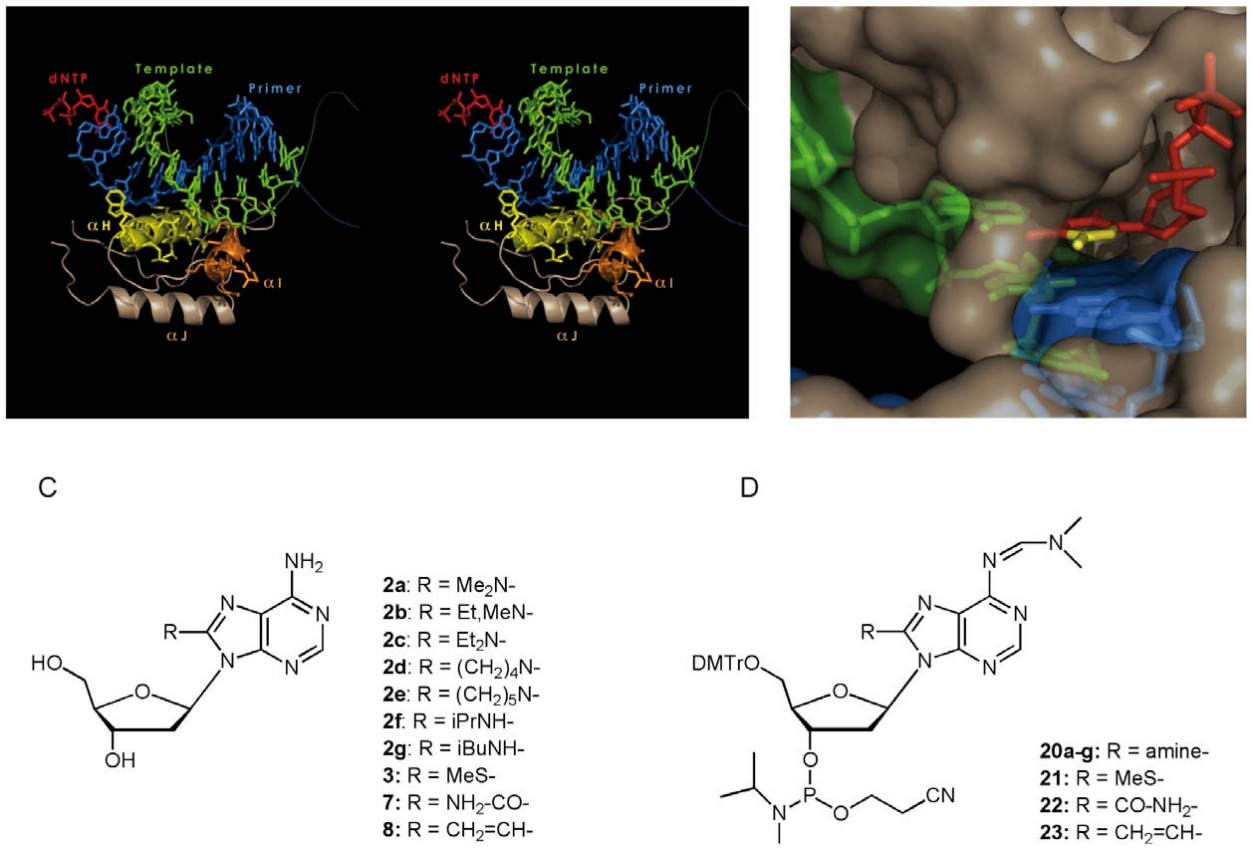
Rationale and nucleos(t)ide analogues used in this study. **A**. Stereoview of the interaction between helices αH and αI of RT thumb domain according to the X-ray structure of HIV-1 RT in complex with a primer•template complex and an incoming dNTP (37). The side chains of helices αH and αI interacting with the minor groove of the primer•template complex are in yellow and orange, respectively; the primer is in blue, the template in green, and the incoming dNTP in red. **B**. Close up of the dNTP binding site. The surface of the p66 subunit of HIV-1 RT is in grey, the template is in green, the primer in blue, and the incoming dTTP is in red, except positions 5 and 6 of the pyrimidine ring which are in yellow. Positions 7 and 8 of an incoming dATP would occupy the same position in the structure. **C**. Nucleoside analogues and **D**. Phosphoramidites used in this study. DMTr is used for dimethoxytrityl group.

The goal of our work was to develop new NRTIs acting as delayed chain terminators (DCTs). To that aim, we sought to introduce modifications on the natural deoxynucleosides that could interfere with the interaction between the primer/template (P/T) complex and helix αH located in the thumb domain of HIV-1 RT (Figure 1A). Indeed, this interaction, 3 to 6 nucleotides downstream of the RT active site is crucial for DNA synthesis [30]. The crystal structure of a P/T•RT•incoming dNTP complex indicates that modifications at position 5 of pyrimidines or position 8 of purines should not prevent incorporation of DCTs into the nascent primer chain, as this face of the incoming nucleotides makes no contact with the RT active site [34] (Figure 1B). Hence, we decided to synthesize and test a series of 2′-deoxyadenosine analogues substituted at position 8 with groups with different organic functions and sizes (Figure 1C). Our DCTs are completely different from traditional NRTIs as they possess an unmodified deoxyribose moiety with a 3′ hydroxyl group. We hypothesize that after incorporation, the modifications will distort the DNA structure and prevent proper interaction with the thumb domain of RT. To test delayed polymerization arrest, we synthesized the phosphoramidite derivatives of the different nucleoside analogues (Figure 1D) and introduced them in DNA oligonucleotides that we used as primers or templates in *in vitro* reverse transcription assays. Finally, we tested the antiviral activity of the nucleoside analogues in cell culture.

### Chemical synthesis

**Figure 2:**
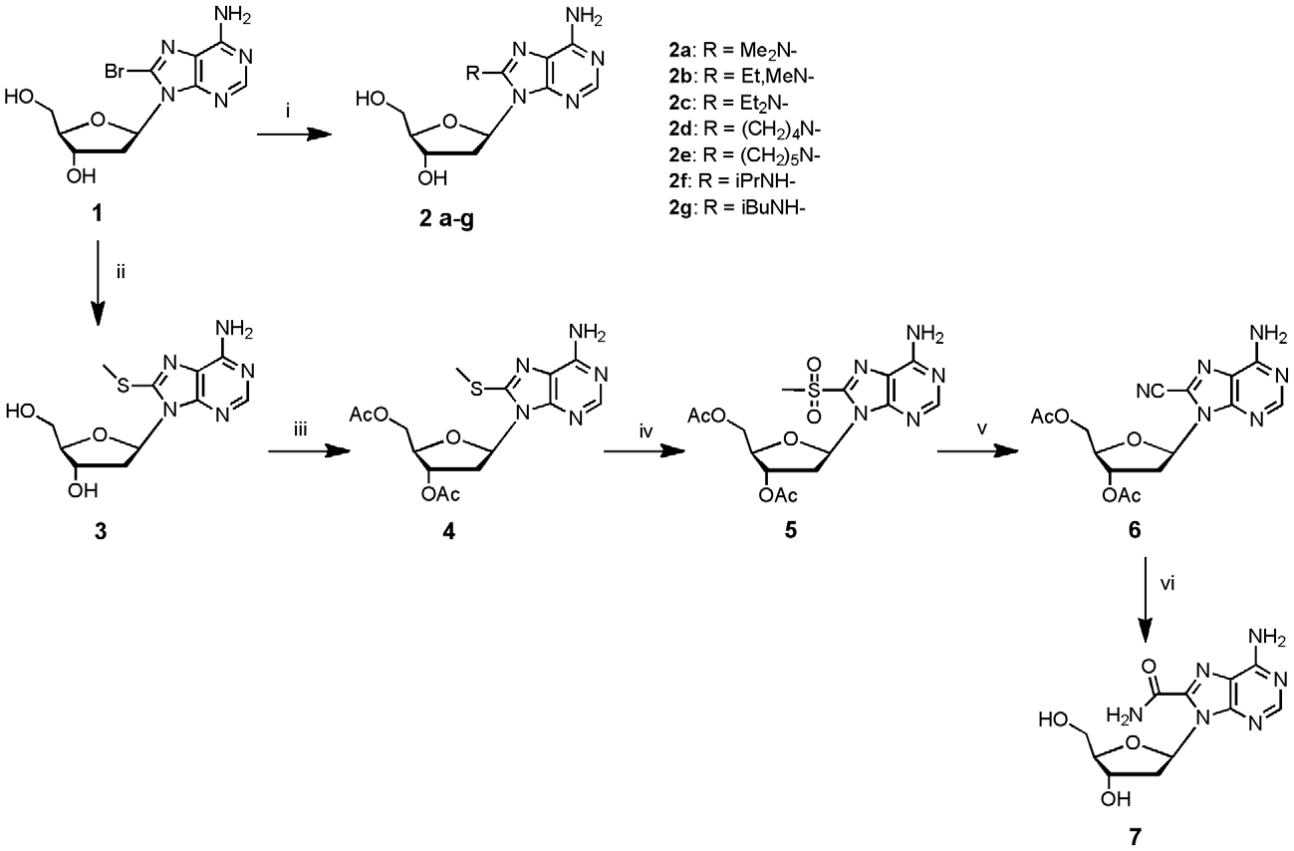
Synthesis of 8-modified nucleoside analogues 2a–g, 3 and 7. (i) Primary amine, MeOH, 65°C, 24 h or secondary amine, MeOH, room temperature, 24 h; (ii) MeSNa 25%/H_2_O, DMF, room temperature, 3 h; (iii) Ac_2_O, pyridine, room temperature, 5 h; (iv) KMnO_4_, CH_3_CO_2_H/H_2_O (50/50 v/v), 0°C, 1 h; (v) NaCN, DMF, room temperature, 3 h; (vi) NaOH 1 M, H_2_O, room temperature, 7 h.

The synthesis of the 8-substituted-2′-dA analogues **2a–g, 3** and **7** was achieved starting from 8-bromo-2′-deoxyadenosine **1** (Figure 2). The nucleophilic displacement of the bromine atom with several primary and secondary amines was performed on the unprotected nucleoside **1** according to the strategy previously described by the group of L.B. Townsend [32]. While preparation of the 8-disubstituted-amino-dA derivatives **2a–e** was performed in methanol at room temperature, the 8-monosubstituted-amino-2′-dA nucleosides **2f–g** required more vigorous conditions, and the reactions were performed at 65°C. The 8-substituted-amino nucleosides **2 a–g** were obtained in moderate to good yields (60 to 97%). The 8-MeS-2′-dA analogue **3** was obtained in 86% yield by treatment of compound **1** with an aqueous solution of sodium methanethiolate in dimethylformamide (DMF). 8-Carbamoyl-2′-dA derivative **7** was obtained starting from the previously synthesized 8-methylthio-2′-dA analogue **3**. Acetylation of the two hydroxyl groups of **3** was performed in quantitative yields using acetic anhydride in pyridine while oxidation of the methylthio group into sulfone was performed with potassium permanganate in acidic conditions. Compound **5** was reacted with sodium cyanate in DMF and gave compound **6** in 80% yield. Removal of the acetyl groups and concomitant hydratation of the cyano function by sodium hydroxide led to compound **7** in 56% yield. 2D-Noesy NMR experiments (Figure S1) showed correlations between modifications introduced on carbon 8 and the H-2′ (up) and H-3′ indicating that our nucleosides exist in the natural anti-conformation. 

**Figure 3:**
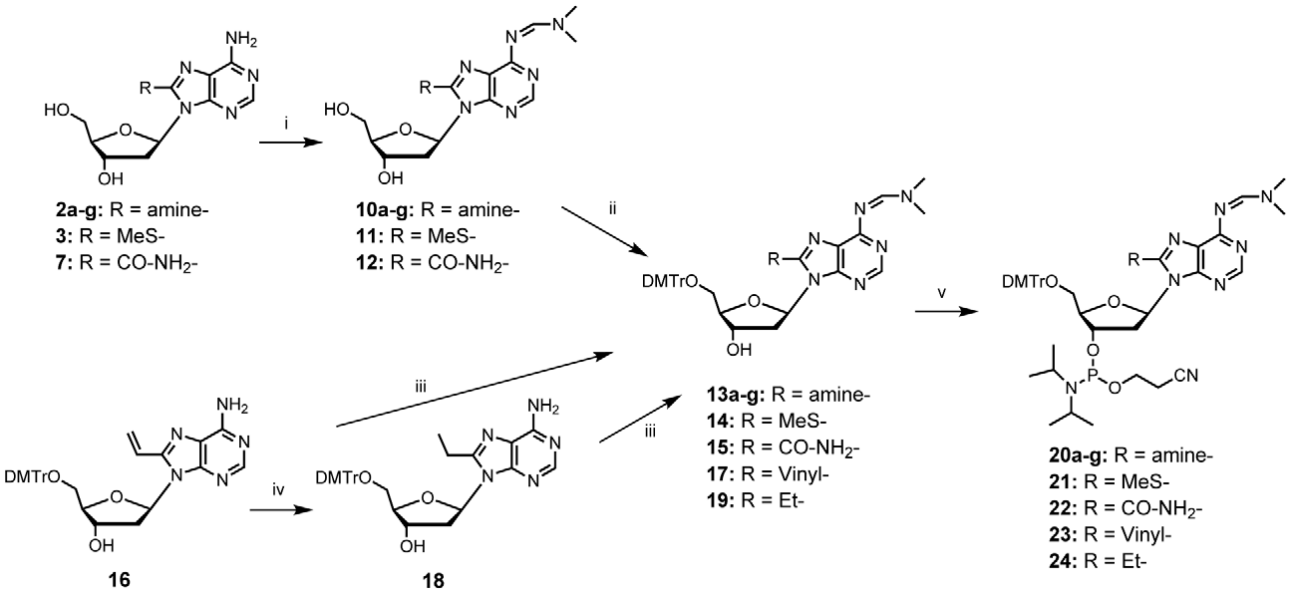
Synthesis of phosphoramidite building blocks 20 a–g, 21, 22, 23 and 24. (i) Me_2_NCH(OMe)_2_, MeOH, room temperature, 17 h; (ii) DMTrCl, pyridine, room temperature, 17 h; (iii) Me_2_NCH(OMe)_2_, DMF, room temperature, 4 h; (iv) H_2_, Pd/C (10%), EtOAc, CH_2_Cl_2_, room temperature, 4 h; (v) (iPr)_2_N(CE)PCl, (iPr)_2_NEt, CH_2_Cl_2_, room temperature, 2.5 h.

Next we synthesized the corresponding phosphoramidites required for incorporation of 8-substituted-2′-deoxyadenosines into oligonucleotides (ODNs) for *in vitro* evaluation on RT. Protection of the exocyclic amino group of compounds **2 a–g, 3** and **7** was achieved with *N,N'*-dimethylformamide-dimethylacetal in methanol (Figure 3). The corresponding base protected nucleosides **10 a–g, 11** and **12** were obtained in 75 to 90% yields. The primary hydroxyl group of these compounds was protected with a dimethoxytrityl group and compounds **13 a–g, 14** and **15** were obtained in good yields varying from 67 to 75%. 8-Vinyl-2′-dA protected nucleoside **17** was prepared from compound **16** according to the protocol previously described by Ben Gaied *et al.* [35]. The synthesis of the 8-ethyl-2′-dA analogue **19** required for the phosphoramidite synthesis was realized straightforward in two steps starting from the known protected 8-vinyl-2′-dA, **16** [35] (Figure 3). Hydrogenation of protected 8-vinyl-2′-dA over palladium catalyst afforded the reduced ethyl intermediate **18** in 94% yield. The amino group of **18** was protected quantitatively with *N,N'*-dimethylformamide dimethylacetal in methanol. The 3′-hydroxyl group of the conveniently protected nucleoside analogues **13 a–g, 14, 15, 17** and **19** was esterified by chloro-cyanoethyl-*N,N'*-diisopropyl-phosphoramidite in dicloromethane in presence of diisopropyl-ethylamine to obtain the corresponding phosphoramidite building block **20a–g, 21, 22, 23** and **24** in yields varying from 70 to 90%.

**Figure 4:**
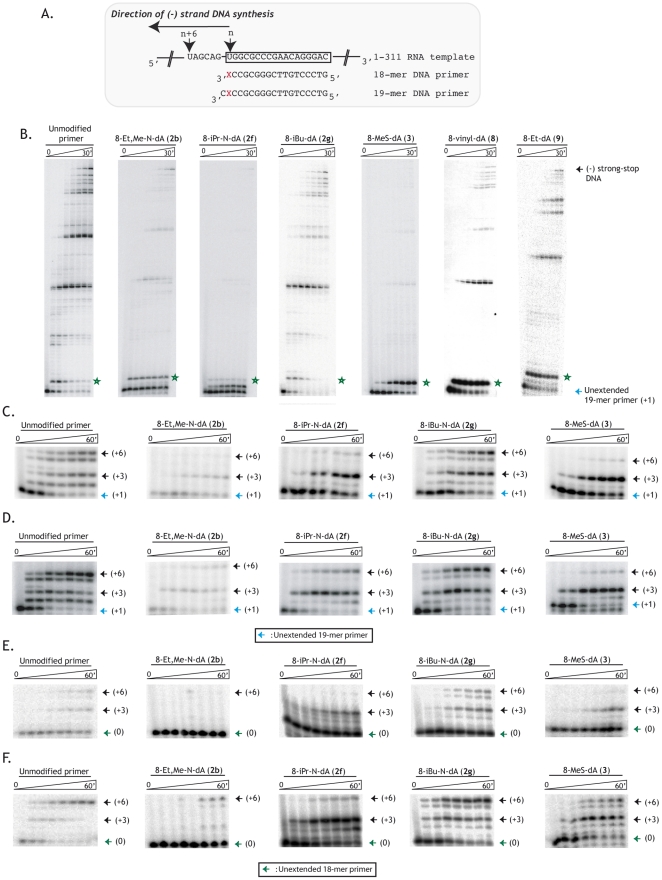
Effect of DCTs on (-) strand DNA synthesis. **A**. Template and primers used for (-) strand DNA synthesis. The template used is 1-311 HIV-1 MAL RNA, for which only part of the sequence is shown. The boxed nucleotides correspond to the Primer Binding Site (PBS). 18- and 19-mer DNA primers were used. X corresponds to either dAMP or a 2′-dA analogue, inserted at the 3′ end (18-mer) or at the penultimate (19-mer) position of the primer. “n” corresponds to the 3′ end of the 18-mer primer strictly complementary to the PBS. **B**. Time course of *in vitro* (-) strand strong-stop DNA synthesis using 19-mer DNA primers containing or not a DCT at the penultimate position. Ten nM of primer/1–311 HIV-1 RNA complexes were pre-incubated with 10 nM of RT and polymerization was initiated by the addition of 50 µM of each of the four dNTPs. Reactions were stopped after 15 and 30 sec, 1, 5, 10, 20 and 30 min. **C./D.** Time course of *in vitro* (−) strand “n+6” DNA synthesis using 19-mer DNA primers containing or not a DCT at the penultimate position. Reaction set-up was the same as previously except that dNTPs were replaced by 20 µM of dTTP, dGTP and dCTP as well as 50 µM of ddATP. In **C.,** 10 nM of RT were used whereas in **D.** 30 nM of RT were used. Reactions were stopped after 30 sec, 1, 5, 10, 20 and 30 min. “+6” refers to the 6^th^ nucleotide to be added with respect to the 5′ end of the PBS sequence. **E./F.** Time course of *in vitro* (−) strand “n+6” DNA synthesis using 18-mer DNA primers containing or not a DCT at the ultimate position. Reaction set-up was the same as previously described for **C./D.** In **E.,** 10 nM of RT were used whereas 30 nM of RT were used in **F**.

**Figure 5:**
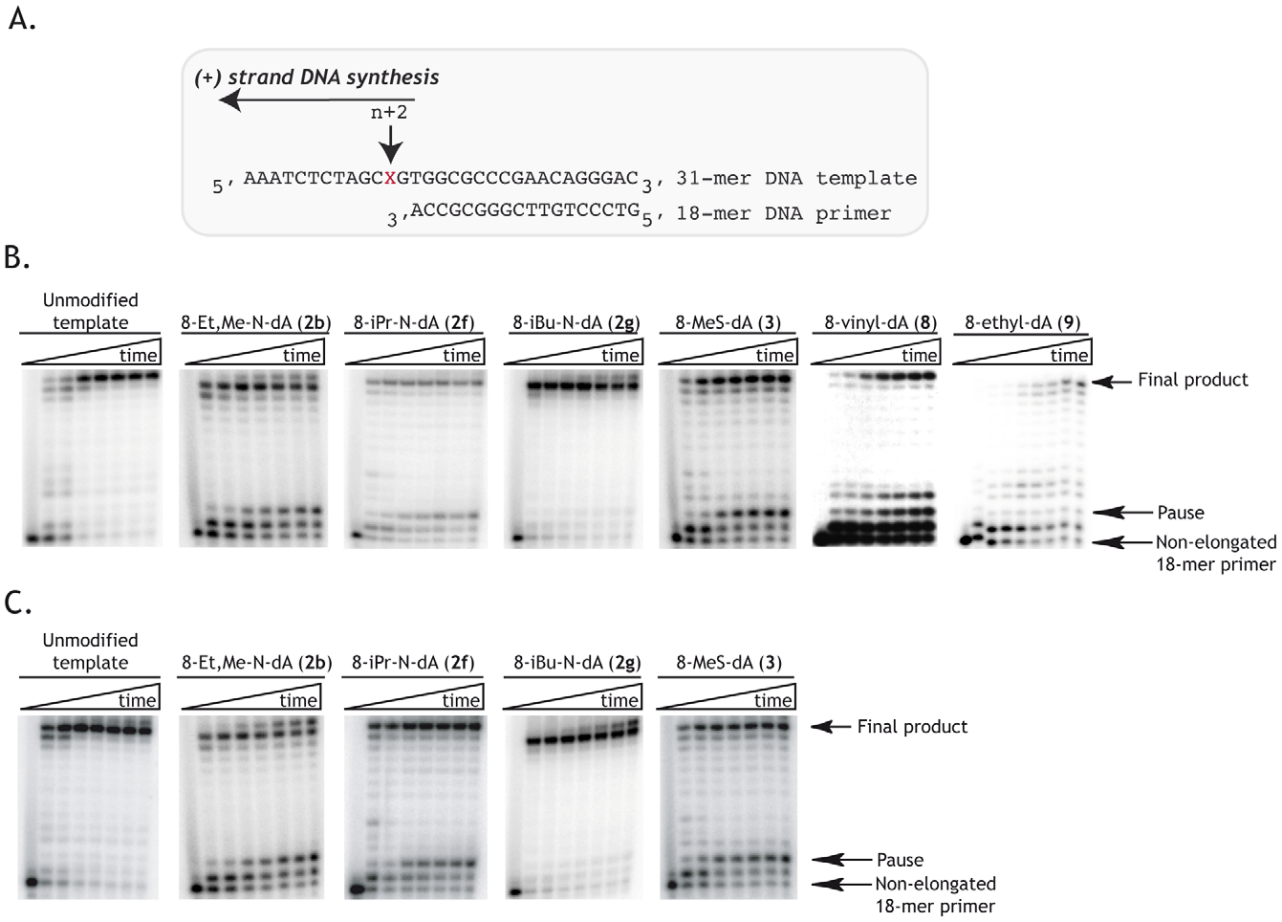
Effects of DCTs on (+) strand DNA synthesis. **A**. Templates and primer used to test the effect of delayed chain terminators on (+) strand DNA synthesis. Synthetic 31-mer DNA oligonucleotides, containing or not the 2′-dA analogues, were used as templates. X corresponds to either dAMP or a 2′-dA analogue. An 18-mer DNA oligonucleotide was used as a primer in this assay. **B**. Time course of *in vitro* (+) strand DNA synthesis when DCTs are inserted in synthetic DNA oligonucleotides that serve as templates. Ten nM of primer/template were pre-incubated with 10 nM of RT and the polymerization reactions were initiated by the addition of 50 µM of each of the four dNTPs. Reactions were stopped after 30 sec, 1, 5, 10, 20, 30 and 60 min. **C.** Reaction set-up was the same as in B. except that 30 nM of RT was used. The percentage of inhibition of DNA synthesis, indicated below each gel, was calculated by comparing the percentage of final product obtained when a DCT is present in the template compared to the percentage of final product obtained with an unmodified template. See also Table 1.

The building blocks **20 a–g**, **21**, **22**, **23** and **24** were used to synthesize the ODN sequences depicted in Figures 4A and 5A. The 18 mer ODNs were prepared on a Universal Support allowing the introduction of the modification at the 3′ end. The cleavage from this support required to be done by ammonia in methanol in dry conditions. Whereas ODNs containing the modifications **2b, 2f–g, 3, 8** and **9** were obtained in satisfying yields, the ones modified with **2a** and **c** were only obtained as traces and we were not able to produce and/or purify ODNs containing modifications **2d–e** and **7**. For this reason *in vitro* experiments were only performed with 6 modified nucleoside analogues.

### 2′-dA analogues induce delayed chain termination

The ability of DCTs to induce chain termination was first estimated by following the *in vitro* synthesis of a 178 nucleotide long (−) strong-stop DNA, the reverse transcription product obtained when a primer is hybridized to the Primer Binding Site (PBS) sequence of the 1–311 HIV-1 MAL RNA. These experiments were performed with a standard 10 nM concentration of HIV-1 RT. The primers we used were 19-mer ODNs, complementary to the PBS extended by one nucleotide at its 5′ end. The primers were either unmodified or modified with a DCT incorporated at the penultimate position of the 19-mers (Figure 4A). In the absence of any modification on the primer, DNA synthesis was processive, with little pausing in the first 36 nucleotides of the (−) strand DNA product and the vast majority of the primer was extended after 5 minutes (Figure 4B). Amongst all the DCTs that were incorporated into DNA primers, only 8-iBu-N-dA (**2g**) gave the same pattern as the unmodified primer (Figure 4B), suggesting that this modified nucleotide behaves like a natural dNTP and does not induce delayed polymerisation arrest. Extension reactions performed with 19-mer DNA primers containing the DCT at the penultimate position from its 3′ end showed that all the other nucleotide analogues tested (**2b**, **2f**, **3**, **8** and **9**) strongly inhibit (−) strong-stop DNA synthesis with, in most cases, a strong pausing site at position +3 (Figure 4B). Quantification of these gels showed that when HIV-1 RT was used at a concentration of 10 nM, incorporation of 8-iPr-N-dA (**2f**) or 8-Et-dA analogue (**9**) in the primer strand inhibited (−) strong-stop DNA synthesis by 48 and 45%, respectively, while the other DCTs were more potent, causing an ∼90% inhibition (Table 1).

**Table 1:** Quantitative analysis of (−) and (+) strand DNA synthesis inhibition.

		Inhibition of (−) strand DNA synthesis (DCT in the primer)					Inhibition of (+) strand DNA synthesis (DCT in the template)	
		DCT at 3′-end-18 mer-		DCT at penultimate position-19 mer-				
		*10 nM RT*	*30 nM RT*	*10 nM RT*		*30 nM RT*	*10 nM RT*	*30 nM RT*
8-Et,Me-N-dA	**2b**	termination	termination	93%*	64%	48%	60%	65%
8-iPr-N-dA	**2f**	81%	90%	48%*	80%	60%	50%	38%
8-iBu-N-dA	**2g**	12%	8%	nd	15%	0%	2%	1%
8-MeS-dA	**3**	77%	64%	88%*	68%	67%	46%	47%
8-vinyl-dA	**8**	nd	nd	90%*	nd	nd	37%	nd
8-Et-dA	**9**	nd	nd	45%*	nd	nd	34%	nd

To estimate inhibition of (−) strand DNA synthesis, DCTs were introduced at the 3′ end or the penultimate position of DNA primers. When inhibition of DNA synthesis was evaluated using the “n+6” DNA synthesis assay, the percentage of inhibition was calculated as follow: *1- (% elongated product “+ DCT”/% elongated product “-DCT”)*. The percentage of elongated product in the presence or absence of DCT was the ratio *“n+6”/(“n+6” + “n+3”)*. Alternatively, the percentage of inhibition was calculated based on inhibition of full-length (−) strand DNA synthesis (*). To study the effect of DCTs on (+) strand DNA synthesis, the nucleoside analogues were incorporated into 31-mer DNA templates. The percentage of inhibition of DNA synthesis was calculated as indicated in the legend of figure 5.

*: percentage of inhibition obtained using the (−) strand strong-stop DNA synthesis assay; nd: not determined.

In order to quantify the inhibitory effects more accurately, we performed +6 DNA synthesis on the same RNA template as above, in the presence of 10 or 30 nM of HIV-1 RT. These experiments were done in the presence of only three of the natural dNTPs (dCTP, dGTP and dGTP), the fourth one being a dideoxy-nucleotide, ddATP. ddATP base-pairs with an uracile that is present in the template, six nucleotides downstream from the position at which the DCT was placed in the elongated primer (Figure 4A). When DCTs were incorporated at the penultimate position of an 19-mer DNA primer, 8-Et,Me-N-dA (**2b**), 8-iPr-N-dA (**2f**) and 8-MeS-dA (**3**) clearly induced a delayed chain termination at both 10 and 30 nM of HIV-1 RT, as evidenced by the accumulation of n+3 products (Figure 4C and 4D), n referring to the position of the DCT (Figure 4A). As expected from the results presented above, 8-iBu-N-dA (**2g**) did not impact (−) strand DNA synthesis. Quantification of the “n+3” and “n+6” products in the absence and presence of modifications allowed us to calculate a percentage of inhibition (see legend to Table 1). When HIV-1 RT was used at a 10 nM concentration, the inhibition varied between 15 to 80% depending on the nature of the DCT. Increasing the RT concentration to 30 nM did not abolish the effect of the active compounds **2b**, **2f** and **3**, but reduced the overall effect of all 4 compounds on (−) strand DNA synthesis, as indicated by the percentage of inhibition which varied from 0 to 67% (Table 1). The same results were obtained when 8-iPr-N-dA (**2f**), 8-iBu-N-dA (**2g**) and 8-MeS-dA (**3**) were located at the last position of an 18-mer DNA primer: at a 10 nM RT concentration, 8-iBu-N-dA (**2g**) had no effect on (−) strand DNA synthesis whereas 8-iPr-N-dA (**2f**) and 8-MeS-dA (**3**) inhibited it by 81 and 77%, respectively. However, with modifications located at the 3′ end of the primer, the increase in RT concentration to 30 nM was less harmful to DNA synthesis than when DCT was placed at the penultimate position. Noticeably 8-Et,Me-N-dA (**2b**) located at the last position of a DNA primer acted as an immediate chain terminator whatever the RT concentration was (Figure 4E and 4F and Table 1). Interestingly, we also found that when DNA rather than RNA was used as a template, 8-MeS-dA (**3**) did not act as a delayed chain terminator (data available in Figure S2). This finding most likely extends to all 2′-dA analogues described in this paper and correlates with the finding that 4′-C-ethyl-2′-dA also blocks DNA synthesis exclusively in the presence of an RNA template, both *in vitro* and *in vivo* [26], [36].

### 2′-dA analogues inhibit (+) strand DNA synthesis

Since delayed polymerization arrest is not complete during (−) strand strong-stop DNA synthesis (Figure 4 and Table 1), we incorporated DCTs into a DNA template and tested their effect on (+) strand DNA synthesis. To that aim, 31-mer DNA oligonucleotides containing or not the DCT analogues (**2b**, **2f**, **2g**, **3**, **8** and **9**) twelve nucleotides from their 5′ end were chemically synthesized (Figure 5A). In the presence of a suitable primer, the DCT analogues face the second nucleotide to be incorporated by RT (Figure 5A), thus allowing for several possible outcomes: (i) the natural dNTP is incorporated opposite the DCT analogue and is extended, leading to normal DNA synthesis; (ii) the presence of the DCT analogue does not allow incorporation of the natural dNTP, mainly generating a primer that is extended by one nucleotide; (iii) the natural dNTP is incorporated but elongation is then blocked or delayed, generating a strong pausing product that reflects the accumulation of a primer extended by 2 nucleotides.

In the presence of an unmodified 31-mer DNA template, extension of the primer is very fast and almost complete within 5vmin, with very little intermediate products (Figure 5B and 5C). The presence of 8-iBu-N-dA (**2g**) in the template does not affect this pattern (Figure 5B and 5C), suggesting that this analogue behaves similarly to its natural counterpart. For all the other nucleoside analogues (8-Et,Me-N-dA (**2b**), 8-iPr-N-dA (**2f**), 8-MeS-dA (**3**), 8-vinyl-dA (**8**) and 8-Et-dA (**9**), enzyme pausing is obvious at positions n+1 and mainly n+2, directly opposite to the DCTs in the template strand, at RT concentrations of 10 or 30 nM (Figure 5B and 5C)). The blockage of DNA synthesis is however not complete and full-length DNA synthesis products are still visible. Overall, these results suggest that not only incorporation of a nucleotide opposite some DCT analogues, but also extension of a duplex terminated by a base-pair involving a DCT analogue are slowed down. The impact of the presence of DCT analogues in the template strand was measured by comparing the percentage of final product in the presence and absence of DCTs. Quantitative data indicated that, for efficient compounds, inhibition of (+) strand DNA synthesis ranges from 34 to 60% at an RT concentration of 10 nM. Increasing the RT concentration to 30 nM did not drastically affect the percentage of inhibition of DNA synthesis which ranged from 38 to 65% (Table 1). These results are in accordance with the ones recently published by Vu *et al.* [26] on 4′-C-ethyl-2′-dA activity.

### One 8-modified-2′-dA analogue reduces HIV-1 replication in cell culture

The *in vitro* experiments we performed indicated that once incorporated into the primer or the template strand, most of our compounds are able to cause delayed termination of reverse transcription. We thus addressed the antiviral activity of our nucleoside analogues in cell culture. All the synthesized nucleoside analogues (**2a–g, 3, 7, 8** and **9**) were tested for their ability to reduce HIV-1 LAI in CEM-SS cells. Viral replication was monitored by measuring the RT activity in the cell culture supernatants, while compound's cytotoxicity on uninfected cells was assessed using an MTT assay [37]. Results are presented in Table 2. One compound (**2f**) displayed a moderate activity (EC_50_ = 14 µM) associated to a slight cytotoxicity (CC_50_ = 81 µM).

**Table 2:** Effect of nucleoside analogues against HIV-1 LAI in CEM-SS cells.

		EC_50_ **µ**M	CC_50_ **µ**M
8-Me_2_-N-dA	**2a**	>100	>100
8-Et,Me-N-dA	**2b**	>100	>100
8-Et_2_-N-dA	**2c**	>100	>100
8-(CH_2_)_4_-N-dA	**2d**	>100	>100
8-(CH_2_)_5_-N-dA	**2 e**	>100	>100
8-iPr-N-dA	**2f**	14.2±1.9	81.3±6.1
8-iBu-N-dA	**2g**	>100	>100
8-MeS-dA	**3**	>100	>100
8-NH_2_-CO-dA	**7**	>100	>100
8-vinyl-dA	**8**	>100	>100
8-Et-dA	**9**	>100	>100

The 50% Effective Concentration (EC_50_) was determined by measuring the RT activity in cell culture supernatants. The 50% Cytotoxic Concentration (CC_50_) on uninfected cells was assessed using an MTT assay. Results are expressed as the mean value ± the standard deviation from four independent experiments.

## Discussion

Deoxyadenosine and deoxycytosine nucleoside analogues locked into North (N) or South (S) conformations have been synthesized previously [18] and tested for their ability to inhibit DNA synthesis at a distance from the polymerization site. Only the N-conformation locked analogues competed with natural dNTPs and were incorporated by HIV-1 RT. They were effective in blocking polymerisation 2 to 3 nucleotides after their incorporation site. Unfortunately, these analogues were not efficiently phosphorylated by cellular kinases and are thus not suitable for further development of anti-HIV-1 drugs. More recently, ETV, which is used to treat hepatitis B virus infections, was shown to be incorporated by HIV-1 RT and to block DNA synthesis at position n+3 (26). In our work, rather than modifying the sugar moiety of the nucleoside, we have chosen to synthesize compounds that are modified on the base. More precisely, eleven analogues of 2′-deoxyadenosine modified on position 8 of the purine ring were designed and chemically synthesized. 2D-Noesy NMR experiments indicated that for 8-iPr-N-dA, 8-MeS-dA or iBu-N-dA the natural anti-conformation is preserved allowing the formation of base pairing and the possible incorporation of the analogues into the nascent viral DNA by RT.

To test the effect of the 2′-dA analogues on (−) strand DNA synthesis, we prepared their phosphoramidite derivatives (Scheme 2) and they were introduced at the 3′ end or the penultimate position of DNA primers by automated solid phase DNA synthesis. Only oligonucleotides modified with compounds **2b**, **2f–g**, **3**, **8** and **9** were obtained with satisfying yields and were used for *in vitro* experiments. Once those primers were annealed to an RNA template, the P/T complexes were elongated by HIV-1 RT in the presence of natural dNTPs. In such an *in vitro* reverse transcription assay, all but one of the 2′-dA analogues inhibited (−) strand DNA synthesis, with effects ranging from 45% to 90% of inhibition (Figure 4 and Table 1). This inhibition is mainly due to strong pausing 2 to 3 nucleotides from the DCT incorporation site (Figure 4). Pausing induced by DCTs does not induce a permanent blockage of reverse transcription at this site, but extension of the paused products is hardly detectable at low (10 nM) RT concentration (Figure 4B). At higher RT concentration (30 nM), extension of the paused products can be detected at the last points of the time course. Remarkably, we found that the 8-iBu-N-dA (**2g**) analogue does not inhibit DNA synthesis at all whereas 8-Et,Me-N-dA (**2b**), which displays delayed polymerisation arrest effects when positioned at the penultimate position of the primer, acts as a direct chain terminator when located at the 3′ end of the primer. Importantly, the overall observed effects during (−) strand DNA synthesis are the predicted ones: DNA synthesis is blocked at a distance from the catalytically active site. Notably, the modifications carried by the adenosine ring are located within the major groove of the P/T duplex, since in most cases HIV-1 RT cannot incorporate nucleoside analogues that are modified on the minor groove interacting site, the latter positions making extensive interactions with the HIV-1 RT catalytic site [34]. Since crystallographic structures of RT•P/T complexes clearly show that interactions of the P/T duplex with αH and αI helices of the thumb domain of HIV-1 RT take place in the minor groove (Figure 1A), the delayed polymerisation outcome that is observed in our experiments must be due to indirect effect(s). Further investigations about the impact of the modifications that we introduced on 2′-dA on the structure of a DNA/DNA duplex, by X-ray crystallography for example, would be of great interest.

Inhibition of (−) strand DNA synthesis was not complete in our *in vitro* assays with any of the nucleoside analogues tested. We therefore set out to test their capacity to inhibit the second round of DNA synthesis, corresponding to (+) strand DNA synthesis once nucleoside analogues have been incorporated into the (−) strand DNA. Inhibition of (+) strand DNA synthesis has already been documented for ETV [27]. With the exception of 8-iBu-N-dA, all nucleoside analogues tested inhibited (+) strand DNA synthesis, with an effect ranging from 34 to 65% of inhibition compared to the situation in the absence of nucleoside analogue (Figure 5 and Table 1).

Hence, all but one of the nucleoside analogues that we conceived to inhibit DNA synthesis by delayed polymerisation arrest effectively act by this mechanism *in vitro*. They also share the additional benefit of displaying a cumulative effect, since they inhibit the synthesis of both (−) and (+) DNA strands.

One of our nucleoside analogues, 8-iPr-N-dA, has a moderate anti-HIV-1 activity in a cell-based assay. The reason why the other compounds are inactive in this assay is presently unknown. It is possible that the phosphorylation steps and/or incorporation of the triphosphorylated analogues by HIV-1 RT are limiting. To investigate the latter hypothesis, testing the incorporation of the triphosphorylated forms of the nucleoside analogues during reverse transcription is crucial. Unfortunately, synthesis and purification of the triphosphorylated forms of the 2′-dA analogues proved to be extremely difficult and could not be achieved in sufficient amount up to now.

On the basis of our *in vitro* assays, a first structure-activity relationship can be established. In the case of the *C*-aminated derivatives, the absence of any delayed chain termination activity for 8-iBu-N-dA (**2g**) compared to 8-iPr-N-dA (**2f**) indicates that the presence of an additional methylene group in the amine introduced at position 8 of the adenine moves the isopropyl group sufficiently away not to disturb the nucleic acid•RT interactions that are necessary for efficient polymerisation. By contrast, the case of 8-Et,Me-N-dA (**2b**) indicates that the presence of two alkyl substitutions on the amine, leading to the presence of a tertiary amine in position 8 of the adenine, inhibits the incorporation of the next incoming dNTP. However, the same substitution, when already embedded into a DNA primer, does inhibit DNA synthesis at a distance from the active site and impairs the incorporation of a natural dNTP when present in the template strand. Importantly, for all the other nucleoside analogues tested, the steric hindrance created by the modifications does not seem to perturb the interactions that are necessary for the incorporation of the next incoming dNTP and the size of the modifications still seems appropriate to interfere with the crucial nucleic acid•RT contact points. At the same time, these modifications also interfere with base pairing of the complementary natural dNTP during (+) strand DNA synthesis. Notably, in the latter case, there is no delayed effect and the blockage of DNA synthesis is immediate, at the modification site. Observation of the crystal structure of a P/T•RT complex [34] reveals that the presence of a modification on position 8 of the purine ring in the template, at the polymerisation site, would most likely create a steric clash with Phe61 and Leu74, within the β3 and β4 sheets of the finger subdomain of HIV-1 RT, thus explaining the immediate arrest of DNA synthesis. Leu74 anchors the template to RT, making it all the more difficult for resistance mutations to be acquired at this position, since any changes would affect the stability of the P/T•RT complex.

In conclusion, we have evidenced a modification site, position 8 of 2′-deoxyadenosine, which induces delayed polymerisation arrest by HIV-1 RT *in vitro.* In addition, 8-iPr-N-dA is the first compound to be described that inhibits viral replication through this particular mechanism and exhibits a relatively low cytotoxic effect for a first generation compound.

One of the main reasons to believe that DCTs are an interesting family of new RT inhibitors to be investigated is their likelihood to escape the resistance pathway that involves excision of the NRTI. This is indeed possible due to the incorporation of a few natural nucleotides before polymerization is stalled. After removal of the last natural nucleotide at the 3′ of the primer, removal of the penultimate nucleotide will compete with re-incorporation of the last one. As excision is negligible when polymerization is possible, RT will never manage to remove 3 or 4 successive nucleotides. Importantly, delayed chain termination reported for the nucleoside analogue ETV protects the elongated primer from excision. Thus, phosphorolytic removal of our DCTs by WT and resistant HIV-1 RT will require further investigation.

Because, unlike NRTIs approved by the Food and Drug Administration (FDA), our DCTs have no modification on the ribose moiety, they should present no cross-resistance with these drugs. More importantly, because of the delayed chain termination mechanism, HIV-1 will be unable to select mutations conferring resistance to DCTs by the existing mechanisms. In addition, as no side chain of the RT active site is in the vicinity of the modifications introduced (Figure 1B), there should be no steric hindrance issues preventing DCT incorporation by HIV-1 RT. Thus, the only way for RT to become resistant will be to select for amino acids in the thumb domain that will accommodate the modifications of DCTs. However, as the primer/template complex moves along the RT, the DCTs will interact with numerous amino acids of the RT thumb, and thus multiple mutations would be required to achieve resistance. In addition, to accommodate the bulky modifications of DCTs, RT would have to select amino acids with small side-chains that will be unable to maintain crucial interactions with the unmodified regions of the primer. Thus, resistance will be difficult to acquire or will be achieved at the prize of a highly reduced viral fitness, giving a chance to the host immune system to control the resistant virus.

## Materials and Methods

### Nucleoside synthesis

Chemical syntheses were carried out using material and manipulations described previously [37]. Amines introduced at position 8 were purchased from Aldrich. They were dried over KOH and distilled before use. UV, mass spectrometry, ^1^H (300 MHz) and ^13^C (75 MHz) NMR data of all compounds are available in Dataset S1.

#### 8-Amino-2′-deoxyadenosine derivatives (2 a–e)

A suspension of 500 mg (1.51 mmol) of 8-bromo-2′-deoxyadenosine[35] (**1**) in methanol (25 ml) was reacted 24 h at room temperature with (30 mmol) of the corresponding amine (**a**: 15 ml of a 2 M solution of dimethylamine in MeOH; **b**: 2.6 ml of ethylmethylamine; **c**: 3.1 ml of diethylamine; **d**: 2.5 ml of pyrrolidine; **e**: 3.0 ml of piperidine). The reaction mixture was evaporated to dryness and the residue was purified by column chromatography (0–10% MeOH in CH_2_Cl_2_) to afford the targeted compounds ((**2a**: 90% yield (400 mg, 1.36 mmol); **2b**: 97% yield (452 mg, 1.46 mmol); **2c**: 85% yield (417 mg, 1.29 mmol); **2d**: 89% yield (430 mg, 1.34 mmol); **2e**: 86% yield (434 mg, 1.30 mmol)).

#### 8-Amino-2′-deoxyadenosine derivatives (2 f–g)

A suspension of 500 mg (1.51 mmol) of 8-bromo-2′-deoxyadenosine (**1**) in methanol (25 ml) was reacted 24 h at 65°C with (30 mmol) of the corresponding amine (**f**: 2.6 ml of isopropylamine; **g**: 3.0 ml of isobutylamine). The reaction mixture was evaporated to dryness and the residue was purified by column chromatography (0–10% MeOH in CH_2_Cl_2_) to afford the targeted compounds ((**2f**: 60% yield (280 mg, 0.91 mmol); **2g**: 68% yield (330 mg, 1.02 mmol)).

#### 8-Methylthio-2′-deoxyadenosine (3)

A solution of 2 g (6.06 mmol) of 8-bromo-2′-deoxyadenosine (**1**) in DMF (2.4 ml) was treated by a 25% aqueous solution of MeSNa (848.5 mg in 3.4 ml H_2_O) and stirred at room temperature for 3 h. The reaction mixture was neutralized by a 1 M solution of HCl. Solvents were eliminated under reduced pressure and crude material was dissolved in hot water. Two batches of crystallization afforded the title compound **3** in 86% yield (1.54 g, 5.19 mmol).

#### 3′,5′-Di-O-acetyl-8-methylthio-2′-deoxyadenosine (4)

A mixture of 1.49 g (5 mmol) of 8-methylthio-2′-deoxyadenosine **3** and 1.88 ml (20 mmol) of acetic anhydride in 25 ml of dry pyridine was stirred at room temperature for 5 h. Water (500 µl) was added, the mixture was evaporated under reduced pressure and co-evaporated successively with toluene, methanol and dichloromethane. Crude material was purified by column chromatography (5–8% MeOH in CH_2_Cl_2_). The title compound was obtained in 97% yield (1.84 g, 4.8 mmol).

#### 3′,5′-Di-O-acetyl-8-methylsulfonyl-2′-deo-xyadenosine (5)

A solution of 1.45 g (3.8 mmol) of compound **4** in an acetic acid/water mixture (50 ml, vol/vol, 50/50) was cooled to 0°C and treated with 1.61 g (10 mmol) of KMnO_4_. The mixture was stirred at 0°C for 45 min and H_2_O_2_ was added until solution decolorized. The resulting mixture was extracted 3 times with CHCl_3_, the combined organic layers were dried over Na_2_SO_4_ and concentrated to dryness. The residue was purified by column chromatography (0–5% MeOH in CH_2_Cl_2_) to give compound **5** in 89% yield (1.40 g, 3.37 mmol).

#### 3′,5′-Di-O-acetyl-8-cyano-2′-deoxyadeno-sine (6)

A mixture of 1.00 g (2.4 mmol) of compound **5** and 152 mg (3.1 mmol) of NaCN in DMF (4.8 ml) was stirred at room temperature for 3 h and neutralized (pH = 7) by addition of a 1 M solution of HCl. The mixture was diluted with water and the compound was extracted once with ethyl acetate. The organic layer was dried over Na_2_SO_4_, concentrated to dryness and purified by column chromatography (0–10% MeOH in EtOAc) to give compound **6** in 80% yield (790 mg, 2.19 mmol).

#### 8-Carbamoyl-2′-deoxyadenosine (7)

A suspension of 600 mg (1.69 mmol) of 3′,5′-di-*O*-acetyl-8-cyano-2′-deoxyadenosine **6** in 35 ml of water was treated with 8.2 ml of a 1 M solution of NaOH. The reaction mixture was stirred at room temperature for 7 h before the neutralization with a Dowex resin (50Wx8 H+). The resin was filtrated off, the solvents were eliminated and the residue was crystallized from water to give pure 8-carbamoyl-2′-deoxy-adenosine with 56% yield (278 mg, 0.94 mmol).

#### 8-Vinyl-2′-deoxyadenosine (8)

Starting from 8-bromo-2′-deoxyadenosine, this compound was prepared according to the procedure previously described [35].

#### 8-Ethyl-2′-deoxyadenosine (9)

Starting from 8-bromo-2′-deoxyadenosine, this compound was prepared according to a procedure previously described [38].

### Phosphoramidites and ODN synthesis

^1^H (300 MHz) and ^13^C (75 MHz) NMR data of compounds are available in Dataset S2.

#### 6-N-Dimethylformamidine-nucleosides (10 a-g, 11 and 12)

About 1.0 mmol of the modified nucleoside (2 a–g, 3 or 7) in methanol (4 ml) was treated with 667 µl (5.0 mmol) of *N,N'*-dimethylformamide-dimethylacetal and stirred overnight at room temperature. The reaction mixture was diluted with ethyl acetate, the organic layer was washed with saturated aqueous NaHCO_3_ and brine, dried over Na_2_SO_4_ and evaporated. Column chromatography (0–10% MeOH in CH_2_Cl_2_) afforded the *N*-6 amino protected nucleosides. Starting quantities, yields and mass analyses are given in Table S1.

#### 5′-O-Dimethoxytrityl-6-N-dimethylforma-midine-nucleosides (13 a–g, 14 and 15)

About 0.8 mmol of nucleoside (**10 a–g, 11** or **12**) in 8 ml of dry pyridine was treated at room temperature with 312 mg (0.92 mmol) of dimethoxytritylchloride. After 17 h the reaction was quenched with water and the solvents were removed under vacuum. The residual oil was dissolved in ethyl acetate and washed successively with saturated aqueous NaHCO_3_, brine and water. The organic layer was dried over Na_2_SO_4_, evaporated to dryness and co-evaporated with toluene, methanol and dichloromethane. The residue was purified by flash silica gel column chromatography (0 – 2% MeOH in CHCl_3_ with 1% Et_3_N) to give the targeted compounds **13 a–g, 14** and **15**. Starting quantities, yields and mass analyses are available in Table S1.

#### 6-N-Dimethylformamidine-5′-O-dimethoxy-trityl-8-vinyl-2′-deoxyadenosine (17)

The synthesis of compound **17** starting from compound **16** and its characteristics are described in [35].

#### 5′-O-Dimethoxytrityl-8-ethyl-2′-deoxyadenosine (18)

150 mg (0.26 mmol) of 8-vinyl-5′-*O*-dimethoxytrityl-2′-deoxyadenosine **16** [35] was introduced in a Schlenk reactor, dissolved in a mixture of EtOAc and CH_2_Cl_2_ and flushed with Ar. before adding a catalytic amount of Pd/C (10%, 0.05 eq.). The reaction mixture was successively and 3 times degassed under vacuum and saturated with H_2_. After 4 h stirring, the reaction mixture was successively and 3 times degassed under vacuum and saturated with Ar. It was then filtered through Celite and diluted with EtOAc. The organic phase was washed with 10 ml of water, saturated aqueous NaHCO_3_ and brine, dried over Na_2_SO_4_ and evaporated to dryness. The ethyl derivative **18** was obtained in 94% yield (141 mg, 0.24 mmol) sufficiently pure to be engaged in the next step without further purification.

#### 6-N-Dimethylformamidine-5′-O-dimethoxy-trityl-8-ethyl-2′-deoxyadenosine (19)

To a solution of 100 mg (0.17 mmol) of compound **18** in 570 µL DMF was added 114 µL (0.86 mmol) of *N,N'*-dimethylformamide-dimethylacetal. After 4 h stirring at room temperature under Argon the reaction was diluted with EtOAc. The organic phase was washed with water, saturated aqueous NaHCO_3_ and brine, dried over Na_2_SO_4_ and evaporated to dryness. The amino protected product **19** was obtained quantitatively (100 mg, 0.17 mmol) sufficiently pure to be engaged in the next step.

#### Preparation of the amidite building blocks 20 a–g, 21, 22, 23 and 24

About 0.5 mmol of starting material dried under reduced pressure for 24 h was dissolved in 7 ml of CH_2_Cl_2_ freshly dried over P_2_O_5_ and distilled. 340 µl (2.00 mmol) of N,N-diisopropylethylamine and 164 µl (0.75 mmol) of chloro-cyanoethyl-*N,N'*-diisopropyl-phosphora-midite were successively added to the solution. After 2.5 h stirring at room temperature, MeOH was added and the reaction was diluted with CH_2_Cl_2_. The organic phase was washed once with water, twice with saturated aqueous NaHCO_3_ and brine, dried over Na_2_SO_4_ and evaporated to dryness. The crude yellow oil was purified by chromatography over silica gel using a mixture of cyclohexane/acetone/NEt_3_ (50∶50∶1) as eluent. The phosphoramidite thus obtained was dissolved in a minimal volume of toluene. The resulting solution was added dropwise to cold hexane (20 volumes, −20°C) to give the targeted phosphoramidite as an amorphous solid. Starting quantities, yields, mass analyses and ^31^P NMR are available in Table S2, for compound **23** data are from Ben Gaied *et al.* [35].

ODN synthesis was performed on an *Applied Biosystem ABI 392* DNA synthesizer using the phosphoramidite chemistry at 1 µmol scale. Solid supports (Universal Support II and Ac-dC-CPG-500) as well as dT, Ac-dC, Pac-dA and iPr-Pac-dG phosphoramidites were purchased from Eurogentec. The standard DNA assembly protocol dimethoxytrityl-off (DMTr-off) was used except for the following modifications. Dichloroacetic acid (3%) in dichloromethane was used for the removal of DMTr protecting group. 5-Ethylthio-1-H-tetrazole was used as activating agent. A longer coupling time (600 s) was used with the modified nucleotide incoming amidites. Phosphite oxidation was done with 2-butanone peroxide in dichloromethane (1 M). ODN was cleaved from the Universal Support by treating with a 2 M ammonia solution in dry methanol for 30 min at room temperature. ODNs were deprotected by treatment with concentrated aqueous ammonia at 60°C for 18 h. ODNs were concentrated to dryness and purified by HPLC on a Dionex DNA-Pac™ PA-100 anion exchange column (9×250 mm) at 60°C with the following gradient system (A = 4 M urea −0.2% acetonitrile −20 mM Mes buffer pH 6.5 −1 mM NaClO_4_ and B = 4 M urea −0.2% acetonitrile −20 mM Mes buffer pH 6.5–400 mM NaClO_4_) from 15 to 70% of solution B in solution A in 50 min with a 1 ml/min flow rate.

Fraction purity was checked by electrophoresis on polyacrylamide gels (20% acrylamide, 8 M urea). The product-containing fractions were pooled and desalted by precipitation in 3 volumes of EtOH with 10% of sodium acetate 3 M. The ODN mass was assigned by Maldi-TOF spectrometry and the final concentration was determined by measuring the absorbance at 260 nm. HPLC retention time and Maldi-TOF mass analysis are available in Table S3.

### Reverse Transcription assays

#### Templates, primers and RTs

Viral RNA, comprising the first 311 nucleotides of the HIV-1 genomic RNA (Mal isolate) was *in vitro* transcribed and purified as previously described [39]. 18-, 19 and 31-mer DNA oligodeoxynucleotides containing the modified 2′-dA analogues respectively at 3′ end, at the penultimate position of 3′ end and at a distance of 12 nucleotides from their 5′ end were chemically synthesized and purified as described above (Figure 5). Unmodified and modified 18- and 19-mer primers were labelled at their 5′ end with [γ-^32^]ATP using phage T4 polynucleotide kinase and purified on 8% denaturing polyacrylamide gels. RNase H (−) reverse transcriptase bearing the E478Q mutation that abolishes RNase H activity (RT^EQ^) [40] was expressed and purified according to a method adapted from [41].

Primer/template (P/T) complexes, at a final concentration of 300 nM, were formed by incubating the primer with a 3 fold excess of either 1–311 viral RNA or modified 31-mer DNA oligonucleotide in water for 2 min at 90°C, cooling on ice for 2 min and incubating for 20 min at 50°C in 100 mM NaCl.

#### Minus strong-stop and “+6” DNA synthesis

Ten nM P/T (with the 311 vRNA as template) were pre-incubated with 10 or 30 nM of RT at 37°C for 4 min. Reactions were initiated by the addition of 50 µM of each of the four dNTPs and stopped at various times with equal amounts of buffer containing formamide. The same procedure was used for synthesis of “+6” DNA except that ddATP was substituted for dATP. Reaction products were denatured for 2 min at 90°C prior to separation on an 8% polyacrylamide denaturing gel and quantified using a Fuji FLA-5100 analyser and the Image Gauge program.

#### Plus-strand DNA synthesis

Thirty one-mer DNA templates, containing or not a modified 2′-deoxyadenosine were hybridized to an 18-mer DNA primer (Figure 5) and subsequent DNA synthesis and analysis of the reaction products were performed as described above.

### HIV-1 inhibition and toxicity of the nucleoside analogues in cell culture

The activity of the nucleoside analogues **2a–g, 3, 7, 8** and **9** on HIV-1 replication was determined by measuring the RT activity associated with virus particles released from CEM-SS cells infected with HIV-1 Lai. The 50% cytotoxic concentration (CC_50_) was evaluated in parallel to the 50% inhibitory concentration (IC_50_)[37].

## Supporting Information

Figure S1 **1D and 2D Noesy spectra of 8-iBu-N-dA and 8-iPr-N-dA.** Experiments were performed in DMSO-d6 as solvent. 1D ^1^H NMR were recorded on a 300 MHz apparatus and 2D Noesy on a 500 MHZ apparatus.
(DOCX)

Figure S2 **Comparative effect of 8-MeS-dA on** (**−) and (+) strand DNA synthesis.** **A**. Templates and primers used for the study. The template is either 1–311 HIV-1 MAL RNA, for which part of the sequence is shown, or a 41-mer DNA oligonucleotide. The primer is a 19- mer DNA strictly complementary to the central part of the template. X corresponds to either dAMP or 8-MeS-dAMP inserted at the penultimate position of the primer. “n” corresponds to the position of the modified nucleotide. **B.** Time course of *in vitro* DNA synthesis using 10 nM of primer/template complexes preincubated with either 10 or 90 nM of HIV-1 RT. Polymerization was initiated by the addition of 20 µM of dTTP, dGTP and dCTP as well as 50 µM of ddATP for the RNA-directed synthesis or 20 µM of dATP, dTTP, dCTP and 50 µM of ddGTP for the DNA directed synthesis. Reactions were stopped after 15, 60 and 120 min. (+6) and (+7) refer to the 6^th^ and 7^th^ nucleotides to be added with respect to the position of the modified nucleotide analogue.
(DOCX)

Dataset S1 **UV, MS, ^1^H and ^13^C NMR of nucleoside analogues.**
(DOCX)

Dataset S2 **^1^H and ^13^C NMR of phosphoramidite building blocks.**
(DOCX)

Table S1 **Analysis of compounds 10a–g, 11, 12, 13a–g, 14 and 15.** Starting quantities, yields and mass analyses.
(DOCX)

Table S2 **Analysis of compounds 20a–g, 21, 22, 23 and 24.** Starting quantities, yields, mass analyses and ^31^P NMR.
(DOCX)

Table S3 **Retention time on anion exchange HPLC column and Maldi-TOF mass of oligodeoxynucleotides.** HPLC was performed on a Dionex DNA-PacTM 28 PA-100 anion exchange column (9×250 mm) at 60°C with the following gradient system (A = 4 M urea 0.2% acetonitrile − 20 mM Mes buffer pH 6.5 − 1 mM NaClO4 and B = 4 M urea − 0.2% acetonitrile − 20 mM Mes buffer pH 6.5 − 400 mM NaClO4) from 15 to 70% of solution B in solution A in 50 min at a 1 ml/min flow rate.
(DOC)
