# Supplementary figures and images for: 8-Modified-2′-Deoxyadenosine Analogues Induce Delayed Polymerization Arrest during HIV-1 Reverse Transcription
Source: PLoS One. 2011 Nov 7;6(11):e27456. doi: 10.1371/journal.pone.0027456 (PMC3210175; doi:10.1371/journal.pone.0027456)

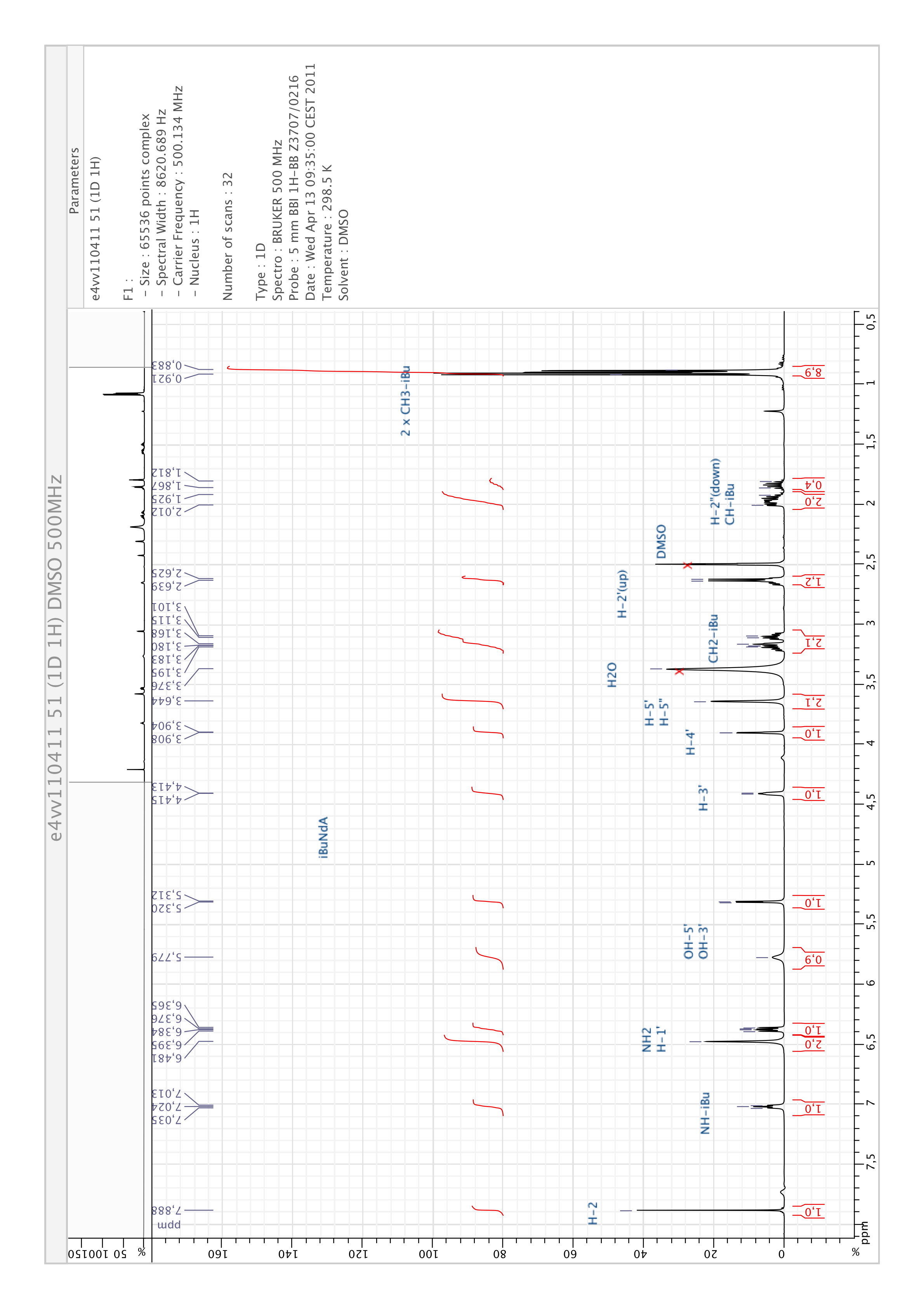


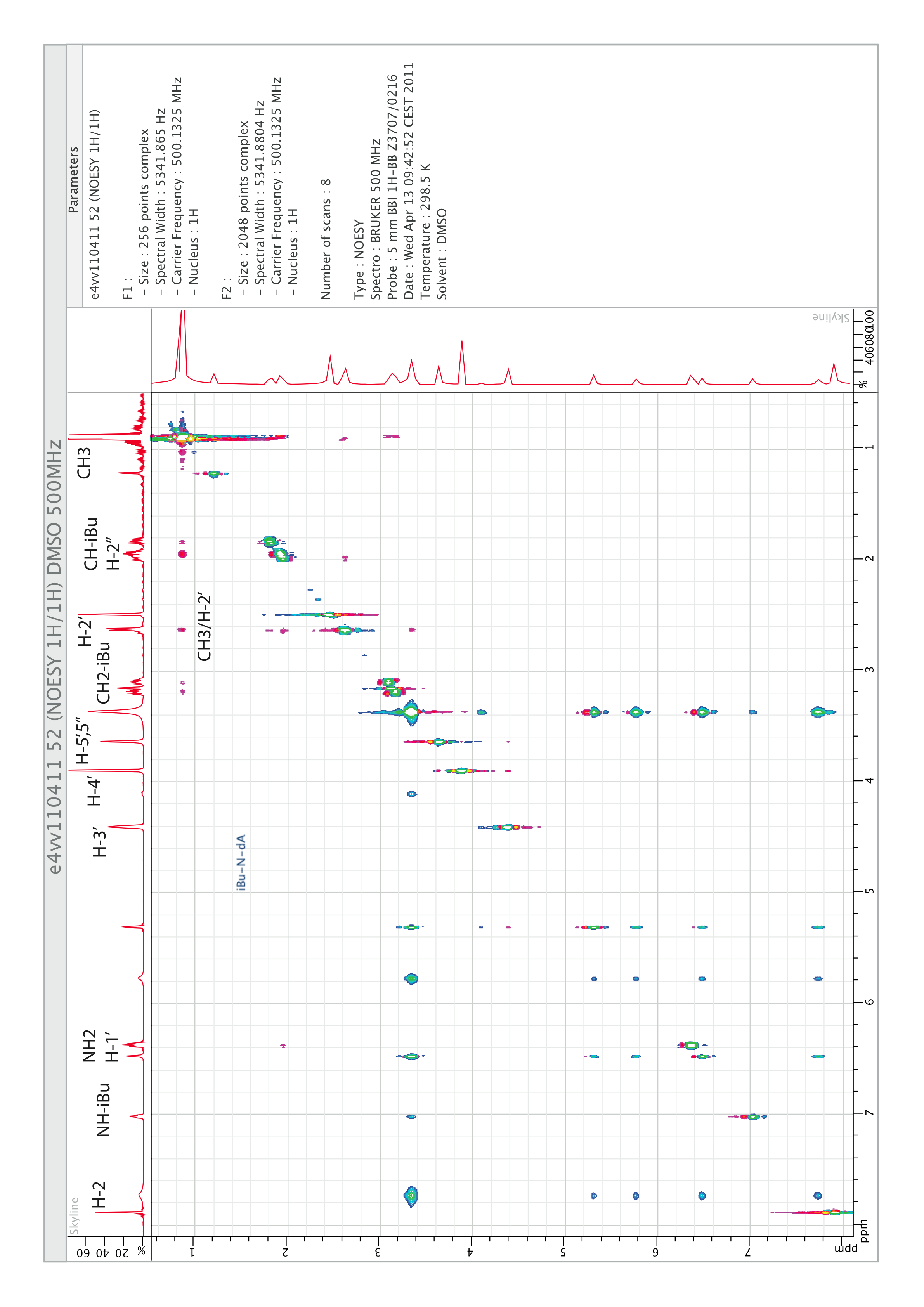


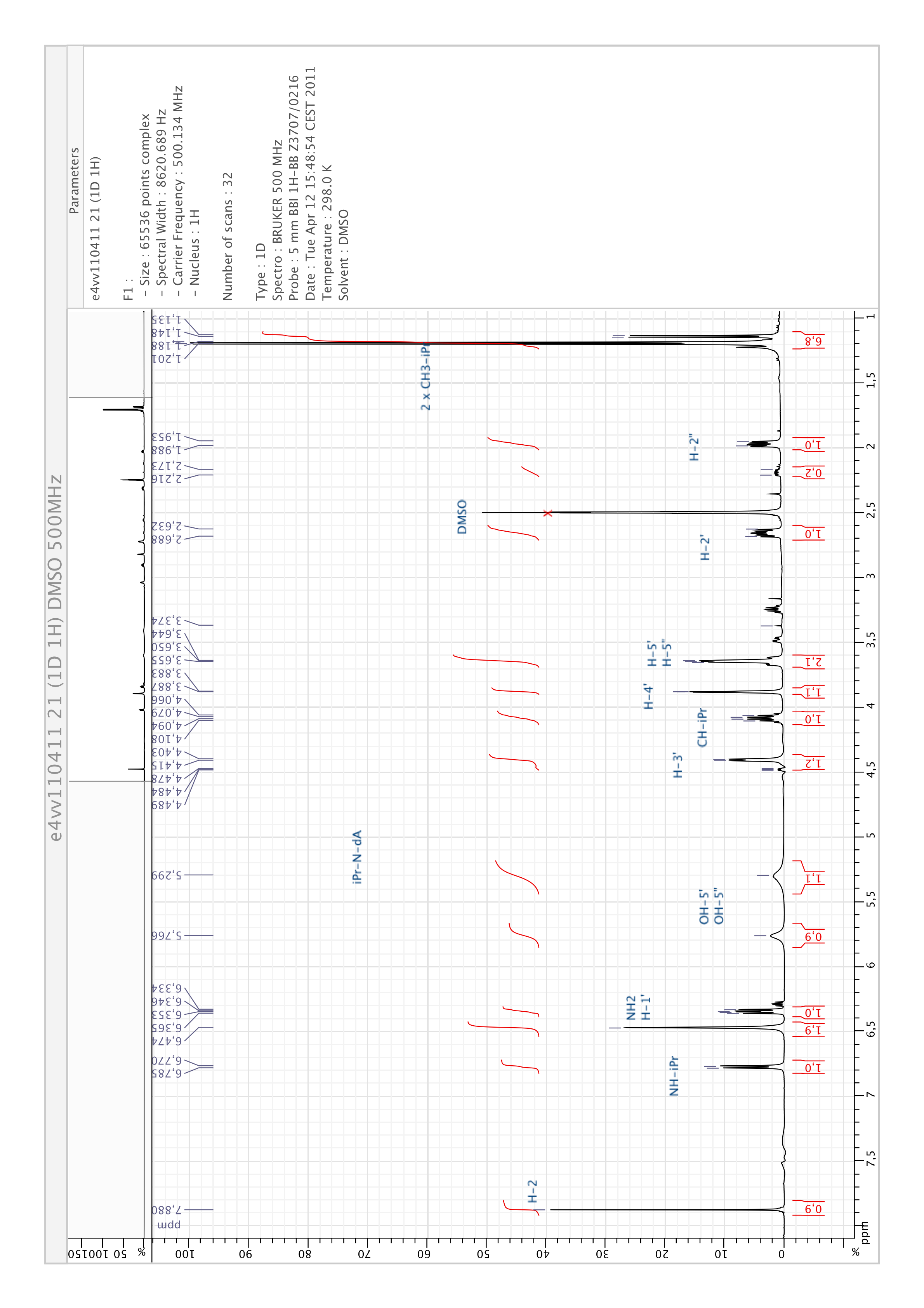

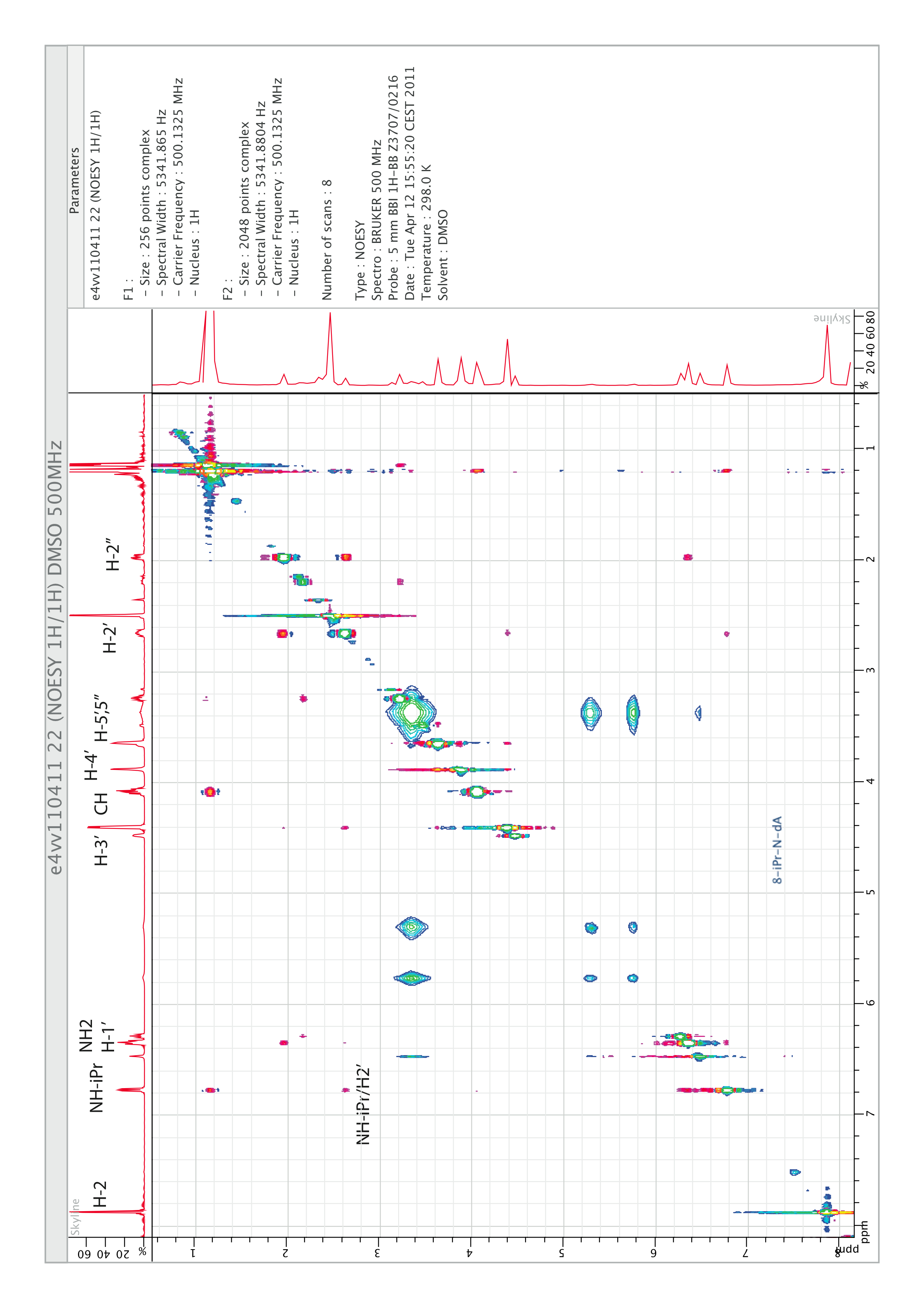

Supplement: Figure S1 — 1D and 2D Noesy spectra of 8-iBu-N-dA and 8-iPr-N-dA. Experiments were performed in DMSO-d6 as solvent. 1D 1H NMR were recorded on a 300 MHz apparatus and 2D Noesy on a 500 MHZ apparatus. (DOCX) [file pone.0027456.s001.docx]

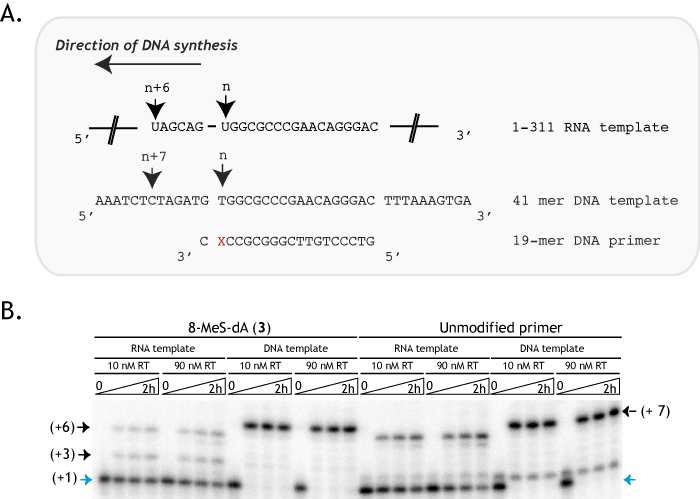

Supplement: Figure S2 — Comparative effect of 8-MeS-dA on (−) and (+) strand DNA synthesis. A. Templates and primers used for the study. The template is either 1–311 HIV-1 MAL RNA, for which part of the sequence is shown, or a 41-mer DNA oligonucleotide. The primer is a 19- mer DNA strictly complementary to the central part of the template. X corresponds to either dAMP or 8-MeS-dAMP inserted at the penultimate position of the primer. “n” corresponds to the position of the modified nucleotide. B. Time course of in vitro DNA synthesis using 10 nM of primer/template complexes preincubated with either 10 or 90 nM of HIV-1 RT. Polymerization was initiated by the addition of 20 µM of dTTP, dGTP and dCTP as well as 50 µM of ddATP for the RNA-directed synthesis or 20 µM of dATP, dTTP, dCTP and 50 µM of ddGTP for the DNA directed synthesis. Reactions were stopped after 15, 60 and 120 min. (+6) and (+7) refer to the 6th and 7th nucleotides to be added with respect to the position of the modified nucleotide analogue. (DOCX) [file pone.0027456.s002.docx]
