# Supplementary material for: 8-Modified-2′-Deoxyadenosine Analogues Induce Delayed Polymerization Arrest during HIV-1 Reverse Transcription
Source: PLoS One. 2011 Nov 7;6(11):e27456. doi: 10.1371/journal.pone.0027456 (PMC3210175; doi:10.1371/journal.pone.0027456)
Supplement: Dataset S1 — UV, MS, 1H and 13C NMR of nucleoside analogues. (DOCX) [file pone.0027456.s003.docx]

**8-Dimethylamino-2'-deoxyadenosine (2a)**

UV (H_2_O): λmax = 279 nm (ε = 14500).

ESI MS (+) (m/z): Calculated for C_12_H_18_N_6_O_3_Li [M+Li]^+^: 301.1600; found: 301.1632.

^1^H NMR (300 MHz, DMSO-*d_6_*): δ = 2.0-2.2 (m, 1H, H-2"), 2.87 (s, 6H, 2 x CH_3_), 3.1-3.2 (m, 1H, H-2'), 3.5-3.7 (m, 2H, H-5' and H-5"), 3.8-3.9 (m, 1H, H-4'), 4.4-4.5 (m, 1H, H-3'), 5.3 (brs, 1H, OH-3'), 5.6-5.7 (m, 1H, OH-5'), 6.13 (dd, 1H, H-1', J_1',2'_ = 7.2 Hz, J_1',2"_ = 5.7 Hz), 7.99 (s, 1H, H-2), 6.94 (s, 2H, NH_2_).

^13^C NMR (75 MHz, DMSO-*d_6_*): δ = 37.4 (C-2'), 43.1 (2 x CH_3_), 62.9 (C-5'), 72.0 (C-3'), 85.3 (C-1'), 88.6 (C-4'), 117.7 (C-5), 149.8 (C-8), 150.6 (C-2), 154.7 (C-4), 156.1 (C-6).

**8-Ethylmethylamino-2'-deoxyadenosine (2b)**

UV (H_2_O): λmax = 276 nm (ε = 14100).

ESI MS (+) (m/z): Calculated for C_13_H_20_N_6_O_3_H [M+H]^+^: 309.1675; found: 309.1694.

^1^H NMR (300 MHz, DMSO-*d_6_*): δ = 1.13 (t, 3H, CH_3_-CH_2_-N, J_CH3,CH2_ = 7.1 Hz), 2.0-2.1 (m, 1H, H-2"), 2.84 (s, 3H, CH_3_-N), 3.1-3.2 (m, 3H, H-2' et CH_2_-N), 3.4-3.7 (2m, 2H, H-5' and H-5"), 3.9-3.8 (m, 1H, H-4'), 4.4-4.5 (m, 1H, H-3'), 5.28 (d, 1H, OH-3', J_OH3',3'_ = 4.3 Hz), 5.6-5.7 (dd, 1H, OH-5', J_OH5',5'_ = 9.1 Hz, J_OH5',5"_ = 3.4 Hz), 6.09 (dd, 1H, H-1', J_1',2'_ = 8.6 Hz, J_1',2"_ = 5.7 Hz), 6.96 (s, 2H, NH_2_), 7.99 (s, 1H, H-2).

^13^C NMR (75 MHz, DMSO-*d_6_*): δ = 12.6 (CH_3_-CH_2_-N), 37.5 (C-2'), 39.1 (CH_3_-N), 49.8 (CH_2_-N), 62.9 (C-5'), 72.0 (C-3'), 85.3 (C-1'), 88.6 (C-4'), 117.4 (C-5), 149.6 (C-8), 150.6 (C-2), 154.7 (C-4), 155.5 (C-6).

**8-Diethylamino-2'-deoxyadenosine (2c)**

UV (H_2_O): λmax = 277 nm (ε = 13900).

ESI MS (+) (m/z): Calculated for C_14_H_22_N_6_O_3_Li [M+Li]^+^: 329.1913; found: 329.1973.

^1^H NMR (300 MHz, DMSO-*d_6_*): δ = 1.06 (t, 6H, 2 x CH_3_-CH_2_-N, J_CH3,CH2_ = 7.0 Hz), 2.0-2.1 (m, 1H, H-2"), 3.0-3.1 (m, 1H, CH-2'), 3.1 (q, 4H, 2 x CH_2_-N, J_CH2,CH3_ = 7.0), 3.5-3.7 (2m, 2H, H-5' and H-5"), 3.8-3.9 (m, 1H, H-4'), 4.4-4.5 (m, 1H, H-3'), 5.28 (d, 1H, OH-3', J_OH3',3'_ = 4.1 Hz), 5.7 (m, 1H, OH-5'), 6.16 (dd, 1H, H-1', J_1',2'_ = 8.2 Hz, J_1',2"_ = 5.8 Hz), 6.99 (s, 2H, NH_2_), 7.99 (s, 1H, H-2).

^13^C NMR (75 MHz, DMSO-*d_6_*): δ = 12.9 (CH_3_-CH_2_-N), 37.7 (C-2'), 46.6 (CH_2_-N), 62.9 (C-5'), 72.1 (C-3'), 85.2 (C-1'), 88.6 (C-4'), 117.5 (C-5), 149.4 (C-8), 150.7 (C-2), 153.7 (C-4), 154.8 (C-6).

**8-Pyrrolidin-2'-deoxyadenosine (2d)**

UV (H_2_O): λmax = 282 nm (ε = 15000).

ESI MS (+) (m/z): Calculated for C_14_H_20_N_6_O_3_H [M+H]^+^: 321.1670; found: 321.1679.

^1^H NMR (300 MHz, DMSO-*d_6_*): δ = 1.8-2.0 (m, 4H, 2 x CH_2_-CH_2_-N), 2.0-2.2 (m, 1H, H-2"), 3.1-3.2 (m, 1H, H-2'), 3.4-3.6 (m, 5H, H-5' & 2 x CH_2_-N), 3.6-3.8 (m, 1H, H-5"), 3.8-3.9 (m, 1H, H-4'), 4.4-4.5 (m, 1H, H-3'), 5.26 (d, 1H, OH-3', J_OH3',3'_ = 4.3 Hz), 5.7-5.8 (m, 1H, OH-5'), 6.19 (dd, 1H, H-1', J_1',2'_ = 6.1 Hz; J_1',2"_ = 8.7 Hz), 6.78 (s, 2H, NH_2_), 7.93 (s, 1H, H-2).

^13^C NMR (75 MHz, DMSO-*d_6_*): δ = 25.5 (2 x CH_2_-CH_2_-N), 37.9 (C-2'), 51.3 (2 x CH_2_-N), 62.9 (C-5'), 72.0 (C-3'), 85.3 (C-1'), 88.6 (C-4'), 118.4 (C-5), 149.6 (C-2), 149.7 (C-8), 154.0 (C-4), 154.4 (C-6).

**8-Piperidin-2'-deoxyadenosine (2e)**

UV (H_2_O): λmax = 275 nm (ε = 13300).

ESI MS (+) (m/z): Calculated for C_15_H_22_N_6_O_3_H [M+H]^+^: 335.1826; found: 335.1842.

^1^H NMR (300 MHz, DMSO-*d_6_*): δ = 1.5-1.8 (2m, 6H, 3 x CH_2_-pip), 2.1-2.2 (m, 1H, H-2"), 3.0-3.3 (m, 5H, H-2' & 2 x CH_2_-N-pip), 3.5-3.8 (m, 2H, H-5' & H-5"), 3.8-3.9 (m, 1H, H-4'), 4.4-4.5 (m, 1H, H-3'), 5.29 (d, 1H, OH-3'), 5.6 (br s, 1H, OH-5'), 6.08 (dd, 1H, H-1', J_1',2'_ = 7.0 Hz; J_1',2"_ = 5.8 Hz), 6.96 (s, 2H, NH_2_), 7.99 (s, 1H, H-2).

^13^C NMR (75 MHz, DMSO-*d_6_*): δ = 24.1 (CH_2_-Nγ-pip), 25.5 (2 x CH_2_-Nβ-pip), 37.4 (C-2'), 52.4 (2 x CH_2_-Nα-pip), 62.9 (C-5'), 72.0 (C-3'), 85.2 (C-1'), 88.6 (C-4'), 117.3 (C-5), 149.6 (C-8), 150.8 (C-2), 154.9 (C-4), 155.5 (C-6).

**8-*i*Propyl-amino-2'-deoxyadenosine (2f)**

UV (H_2_O): λmax = 277 nm (ε = 14200).

ESI MS (+) (m/z): Calculated for C_13_H_20_N_6_O_3_H [M+H]^+^: 309.1675; found: 309.1798.

^1^H NMR (300 MHz, DMSO-*d_6_*): δ = 1.9-2.0 (m, 1H, H-2"), 2.6-2.8 (m, 1H, H-2'), 3.6-3.7 (m, 2H, H-5' and 5"), 3.8-3.9 (m, 1H, H-4'), 4.0-4.1 (m, 1H, CH-iPr), 4.4-4.5 (m, 1H, H-3'), 5.28 (d, 1H, OH-3', J_OH3',3'_ = 3.6 Hz), 5.75 (t, 1H, OH-5', J_OH5',5'_ = J_OH5',5"_ = 4.8 Hz), 6.34 (dd, 1H, H-1', J_1',2'_ = 9.0 Hz, J_1',2"_ = 5.8 Hz), 6.46 (s, 2H, NH_2_), 6.77 (d, 1H, NH, J_NH,CH-iPr_ = 7.7 Hz), 7.88 (s, 1H, H-2).

^13^C NMR (75 MHz, DMSO-*d_6_*): δ = 22.9 and 23.2 (2 x CH_3_-iPr), 37.9 (C-2'), 44.3 (CH-N-iPr), 62.1 (C-5'), 71.8 (C-3'), 83.4 (C-1'), 87.9 (C-4'), 117.5 (C-5), 148.8 (C-2), 149.7 (C-8), 151.0 (C-4), 152.6 (C-6).

**8-*i*Butyl-amino-2'-deoxyadenosine (2g)**

UV (H_2_O): λmax = 278 nm (ε = 14700).

ESI MS (+) (m/z): Calculated for C_14_H_22_N_6_O_3_Na [M+Na]^+^: 345.1651; found: 345.1684.

^1^H NMR (300 MHz, DMSO-*d_6_*): δ = 0.8-1.0 (m, 6H, 2 x CH_3_-iBut), 1.9-2.0 (m, 2H, H-2" and CH-iBut), 2.6-2.7 (m, 1H, H-2'), 3.1-3.3 (m, 2H, CH_2_-iBut), 3.6-3.7 (m, 2H, H-5' and H-5"), 3.8-3.9 (m, 1H, H-4'), 4.4-4.5 (m, 1H, H-3'), 5.30 (d, 1H, OH-3', J_OH3',3'_ = 3.7 Hz), 5.77 (t, 1H, OH-5', J_OH5',5'_ = J_OH5',5"_ = 4.0 Hz), 6.37 (dd, 1H, H-1', J_1',2'_ = 9.4 Hz, J_1',2"_ = 5.7 Hz), 6.45 (s, 2H, NH_2_), 6.99 (t, 1H, NH, J_NH,CH2-iBut_ = 5.5 Hz), 7.88 (s, 1H, H-2).

^13^C NMR (75 MHz, DMSO-*d_6_*): δ = 20.1 and 20.7 (2 x CH_3_-iBut), 27.7 (CH-iBut), 38.1 (C-2'), 50.3 (CH_2_-N-iBut), 62.0 (C-5'), 71.9 (C-3'), 83.4 (C-1'), 87.8 (C-4'), 117.3 (C-5), 148.9 (C-2), 149.8 (C-8), 151.6 (C-4), 152.6 (C-6).

**8-Methylthio-2'-deoxyadenosine (3)**

UV (H_2_O): λmax = 279 nm (ε = 13400).

ESI MS (+) (m/z): Calculated for C_11_H_15_N_5_O_3_SNa [M+Na]^+^: 320.0793; found: 320.0918.

^1^H NMR (300 MHz, DMSO-*d_6_*): δ = 2.1-2.2 (m, 1H, H-2'), 2.72 (s, 3H, CH_3_-S), 3.0-3.2 (m, 1H, H-2"), 3.5-3.7 (2m, 2H, H-5' and H-5"), 3.8-3.9 (m, 1H, H-4'), 4.4-4.5 (m, 1H, H-3'), 5.3-5.4 (2m, 2H, OH-5' and OH-3'), 6.21 (t, 1H, H-1', J_1',2'_ = J_1',2"_ = 7.3 Hz), 7.2 (sl, 2H, NH_2_), 8.06 (s, 1H, H-2).

^13^C NMR (75 MHz, DMSO-*d_6_*): δ = 14.9 (CH_3_-S), 37.8 (C-2'), 62.7 (C-5'), 71.7 (C-3'), 85.2 (C-1'), 88.7 (C-4'), 120.0 (C-5), 149.6 (C-4), 151.2 (C-8), 151.6 (C-2), 154.8 (C-6).

**3',5'-Di-*O*-acetyl-8-methylthio-2'-deoxyadenosine (4)**

ESI MS (+) (m/z): Calculated for C_15_H_19_N_5_O_5_SNa [M+Na]^+^: 404.1005; found: 404.0976.

^1^H NMR (300 MHz, DMSO-*d_6_*): δ = 1.97 & 2.10 (2s, 6H, 2 x CH_3_-CO), 2.4-2.5 (m, 1H, H-2"), 2.72 (s, 3H, CH_3_-S), 3.5-3.6 (m, 1H, H-2'), 4.1-4.2 (m, 2H, H-5' & H-5"), 4.3-4.4 (m, 1H, H-4'), 5.4-5.6 (m, 1H, H-3'), 6.26 (t, 1H, H-1', J_1',2'_ = J_1',2"_ = 6.8 Hz), 7.2 (sl, 2H, NH_2_), 8.10 (s, 1H, H-2).

^13^C NMR (75 MHz, DMSO-*d_6_*): δ = 14.9 (CH_3_-S), 20.9 & 21.2 (2 x CH_3_-CO), 34.1 (C-2'), 63.8 (C-5'), 74.4 (C-3'), 81.8 (C-1'), 84.3 (C-4'), 120.0 (C-5), 149.7 (C-4), 151.5 (C-8), 152.0 (C-2), 154.7 (C-6), 170.6 (2 x CO-CH_3_).

**3',5'-Di-*O*-acetyl-8-methylsulfonyl-2'-deoxyadenosine (5)**

ESI MS (+) (m/z): Calculated for C_11_H_15_N_5_O_7_SNa [M+Na]^+^: 436.0903; found: 436.0885.

^1^H NMR (300 MHz, DMSO-*d_6_*): δ = 1.93 & 2.09 (2s, 6H, 2 x CH_3_-CO), 2.5-2.6 (m, 1H, H-2"), 3.58 (s, 3H, CH_3_-SO_2_), 3.6-3.7 (m, 1H, H-2'), 4.2-4.3 (m, 2H, H-5' & H-5"), 4.4-4.5 (m, 1H, H-4'), 5.5-5.6 (m, 1H, H-3'), 6.85 (t, 1H, H-1', J_1',2'_ = J_1',2"_ = 6.6 Hz), 7.9 (sl, 2H, NH_2_), 8.29 (s, 1H, H-2).

^13^C NMR (75 MHz, DMSO- *d_6_*): δ = 20.9 & 21.3 (2 x CH_3_-CO), 34.9 (C-2'), 43.6 (CH_3_-SO_2_), 63.8 (C-5'), 74.5 (C-3'), 82.5 (C-1'), 85.3 (C-4'), 118.2 (C-5), 146.0 (C-4), 150.4 (C-8), 155.5 (C-2), 157.7 (C-6), 170.5 (2 x CO-CH_3_).

**3',5'-Di-*O*-acetyl-8-cyano-2'-deoxyadenosine (6)**

ESI MS (+) (m/z): Calculated for C_15_H_16_N_6_O_5_H [M+H]^+^: 361.1260; found: 361.1270.

^1^H NMR (300 MHz, DMSO-*d_6_*): δ = 1.99 & 2.11 (2s, 6H, 2 x CH_3_-CO), 2.6-2.7 (m, 1H, H-2"), 3.4-3.5 (m, 1H, H-2'), 4.1-4.4 (m, 1H, H-5"), 4.3-4.4 (m, 2H, H-4' & H-5'), 5.4-5.6 (m, 1H, H-3'), 6.45 (t, 1H, H-1', J_1',2'_ = J_1',2"_ = 6.6 Hz), 7.9-8.1 (m, 2H, NH_2_), 8.32 (s, 1H, H-2).

^13^C NMR (75 MHz, DMSO- *d_6_*): δ = 20.8 & 21.2 (2 x CH_3_-CO), 35.6 (C-2'), 63.5 (C-5'), 74.1 (C-3'), 82.5 (C-1'), 85.4 (C-4'), 111.8 (CN), 120.0 (C-5), 137.6 (C-8), 149.2 (C-4), 156.2 (C-2), 157.3 (C-6), 170.5 (2 x CO-CH_3_).

**8-Carbamoyl-2'-deoxyadenosine (7)**

UV (H_2_O) λmax = 290 nm (ε = 9700).

ESI MS (+) (m/z): Calculated for C_11_H_14_N_6_O_4_Na [M+Na]^+^: 317.0974; found: 317.0986.

^1^H NMR (300 MHz, DMSO-*d_6_*): δ = 1.9-2.0 (m, 1H, H-2"), 2.0-2.2 (m, 1H, H-2'), 3.5-3.6 (m, 2H, H-5' & H-5"), 3.7-3.8 (m, 1H, H-4'), 4.1-4.2 (m, 1H, H-3'); 4.94 (t, 1H, OH-5', J_OH5',5'_ = J_OH5',5"_ = 5.5 Hz); 5.19 (d, 1H, OH-3', J_OH3',3'_ = 4.3 Hz), 6.15 (t, 1H, H-1', J_1',2'_ = J_1',2"_ = 6.7 Hz), 6.7-6.9 (m, 1H, NH_2_-amine), 7.03 (s, 1H, NH_2_-amide), 7.1-7.3 (m, 1H, NH_2_-amine), 7.39 (s, 1H, NH_2_-amide), 7.60 (s, 1H, H-2).

^13^C NMR (75 MHz, DMSO- *d_6_*): δ = 38.9 (C-2'), 62.8 (C-5'), 71.9 (C-3'), 86.1 (C-1'), 88.9 (C-4'), 118.5 (C-5), 142.4 (C-8), 150.3 (C-4), 153.7 (C-2), 157.4 (C-6), 161.4 (CO-amide).
