# Supplementary material for: 8-Modified-2′-Deoxyadenosine Analogues Induce Delayed Polymerization Arrest during HIV-1 Reverse Transcription
Source: PLoS One. 2011 Nov 7;6(11):e27456. doi: 10.1371/journal.pone.0027456 (PMC3210175; doi:10.1371/journal.pone.0027456)
Supplement: Dataset S2 — 1H and 13C NMR of phosphoramidite building blocks. (DOCX) [file pone.0027456.s004.docx]

**8-Dimethylamino-6-*N,N*-dimethylformamidine-2'-dA** (**10a)**

^1^H NMR (300 MHz, DMSO-*d_6_*): δ = 2.0-2.2 (m, 1H, H-2"), 2.84 (s, 6H, 2 x CH_3_-N), 3.05 (s, 3H, CH_3_-N), 3.1-3.2 (m, 4H, H-2' & CH_3_-N), 3.5-3.6 (m, 2H, H-5' & H-5"), 3.8-3.9 (m, 1H, H-4'), 4.4-4.5 (m, 1H, H-3'), 5.4 (br s, 1H, OH-3'), 5.7 (br s, 1H, OH-5'), 6.09 (dd, 1H, H-1', J_1',2'_ = 7.1 Hz, J_1',2"_ = 5.6 Hz), 7.79 (s, 1H, H-2), 8.81 (s, 1H, CH=N).

^13^C NMR (75 MHz, DMSO-*d_6_*): δ = 35.7 (2 x CH_3_-N), 37.5 (C-2'), 41.5 (2 x CH_3_-N), 61.7 (C-5'), 71.2 (C-3'), 80.2 (C-1'), 88.9 (C-4'), 123.4 (C-5), 149.8 (C-2), 151.0, 151,5 & 152.7 (C-4, C-6 & C-8), 159.8 (CH=N).

**6-*N,N*-Dimethylformamidine-8-ethylmethylamino-2'-dA** (**10b)**

^1^H NMR (300 MHz, DMSO-*d_6_*): δ = 1.08 (t, 3H, CH_3_-CH_2_-N, J_CH3,CH2_ = 7.0 Hz), 2.0-2.1 (m, 1H, H-2"), 2.81 (s, 3H, CH_3_-N), 3,09 & 3.15 (2s, 6H, 2 x CH_3_-N), 3.2-3.3 (m, 3H, H-2' & CH_2_-N), 3.4-3.6 (2m, 2H, H-5' & H-5"), 3.8-3.9 (m, 1H, H-4'), 4.4-4.5 (m, 1H, H-3'), 5.16 (d, 1H, OH-3', J_OH3',3'_ = 4.4 Hz), 5.7 (m, 1H, OH-5'), 6.00 (dd, 1H, H-1', J_1',2'_ = 8.8 Hz, J_1',2"_ = 5.6 Hz), 7.91 (s, 1H, H-2), 8.83 (s, 1H, CH=N).

^13^C NMR (75 MHz, DMSO-*d_6_*): δ = 12.6 (CH_3_-CH_2_-N), 35.7 & 36.2 (3 x CH_3_-N), 37.5 (C-2'), 51.2 (CH_2_-N), 62.8 (C-5'), 71.9 (C-3'), 85.2 (C-1'), 88.5 (C-4'), 125.2 (C-5), 148.7 (C-2), 152.6, 155.5 & 155.9 (C-4, C-6 & C-8), 157.6 (CH=N).

**8-Diethylamino-6-*N,N-*dimethylformamidine-2'-dA** (**10c)**

^1^H NMR (300 MHz, DMSO-*d_6_*): δ = 1.08 (t, 6H, 2 x CH_3_-CH_2_-N, J_CH3,CH2_ = 7.0 Hz), 2.0-2.1 (m, 1H, H-2"), 3.11 & 3.17 (2s, 6H, 2 x CH_3_-N), 3.2-3.3 (m, 5H, 2 x CH_2_-N & H-2'), 3.5-3.8 (2m, 2H, H-5' & H-5"), 3.8-3.9 (m, 1H, H-4'), 4.4-4.5 (m, 1H, H-3'), 5.29 (d, 1H, OH-3', J_OH3',3'_ = 3.9 Hz), 5.5-5.6 (m, 1H, OH-5'), 5.76 (dd, 1H, H-1', J_1',2'_ = 7.9 Hz, J_1',2"_ = 5.8 Hz), 8.25 (s, 1H, H-2), 8.80 (s, 1H, CH=N).

^13^C NMR (75 MHz, DMSO-*d_6_*): δ = 13.0 (CH_3_-CH_2_-N), 35.0 (2 x CH_3_-N), 38.7 (C-2'), 46.3 (2 x CH_2_-N), 62.9 (C-5'), 72.0 (C-3'), 85.2 (C-1'), 88.5 (C-4'), 124.2 (C-5), 149.8 (C-2), 152.3 & 154.8 (C-4 & C-8), 157.7 (C-6), 157.9 (CH=N).

**8-Pyrrolidin-6-N,N-diméthylformamidine-2'-dA** (**10d)**

^1^H NMR (300 MHz, DMSO-*d_6_*): δ = 1.8-1.9 (m, 4H, 2 x CH_2_-CH_2_-N, J_CH3,CH2_ = 7.0 Hz), 2.1-2.2 (m, 1H, H-2"), 3.10 & 3.15 (2s, 6H, 2 x CH_3_-N), 3.3-3.5 (m, 5H, 2 x CH_2_-N & H-2'), 3.6-3.8 (m, 2H, H-5' & H-5"), 3.8-3.9 (m, 1H, H-4'), 4.4-4.5 (m, 1H, H-3'), 5.25 (d, 1H, OH-3', J_OH3',3'_ = 4.1 Hz), 5.6-5.7 (m, 1H, OH-5'), 5.98 (dd, 1H, H-1', J_1',2'_ = 8.4 Hz, J_1',2"_ = 6.0 Hz), 8.05 (s, 1H, H-2), 8.75 (s, 1H, CH=N).

^13^C NMR (75 MHz, DMSO-*d_6_*): δ = 26.0 (2 x CH_2_-CH_2_-N), 35.0 (2 x CH_3_-N), 38.2 (C-2'), 49.5 (2 x CH_2_-N), 62.9 (C-5'), 72.0 (C-3'), 85.2 (C-1'), 88.5 (C-4'), 120.5 (C-5), 149.6 (C-2), 150.2 & 154.0 (C-4 & C-8), 154.6 (C-6), 157.6 (CH=N).

**8-Piperidin-6-N,N-diméthylformamidine-2'-dA** (**10e)**

^1^H NMR (300 MHz, DMSO-*d_6_*): δ = 1.6-1.8 (m, 6H, 3 x CH_2_-pip), 2.0-2.1 (m, 1H, H-2"), 3.09 & 3.14 (2s, 6H, 2 x CH_3_-N), 3.1-3.3 (m, 5H, 2 x CH_2_-N & H-2'), 3.5-3.8 (m, 2H, H-5' & H-5"), 3.8-3.9 (m, 1H, H-4'), 4.4-4.5 (m, 1H, H-3'), 5.18 (d, 1H, OH-3', J_OH3',3'_ = 4.1 Hz), 5.5-5.6 (m, 1H, OH-5'), 5.96 (dd, 1H, H-1', J_1',2'_ = 7.2 Hz, J_1',2"_ = 5.9 Hz), 8.05 (s, 1H, H-2), 8.85 (s, 1H, CH=N).

^13^C NMR (75 MHz, DMSO-*d_6_*): δ = 24.4 (CH_2_-Nγ-pip), 25.5 (2 x CH_2_-Nβ-pip), 35.0 (2 x CH_3_-N), 38.7 (C-2'), 49.3 (2 x CH_2_-N), 62.8 (C-5'), 72.3 (C-3'), 85.0 (C-1'), 88.5 (C-4'), 121.2 (C-5), 149.8 (C-2), 151.3 & 154.8 (C-4 & C-8), 155.7 (C-6), 157.9 (CH=N).

**8-*i*Propyl-amino-6-*N,N-*dimethylformamidine-2'-dA** (**10f)**

^1^H NMR (300 MHz, DMSO-*d_6_*): δ = 1.14 & 1.16 (2s, 6H, 2 x CH_3_-iPr), 1.9-2.0 (m, 1H, H-2"), 2.6-2.8 (m, 1H, H-2'), 3.02 & 3.11 (2s, 6H, 2 x CH_3_-N), 3.6-3.7 (m, 2H, H-5' & 5"), 3.8-4.0 (2m, 2H, H-4' & CH-iPr), 4.4-4.5 (m, 1H, H-3'), 5.34 (d, 1H, OH-3', J_OH3',3'_ = 3.5 Hz), 5.75 (t, 1H, OH-5', J_OH5',5'_ = J_OH5',5"_ = 4.6 Hz), 6.29 (dd, 1H, H-1', J_1',2'_ = 8.7 Hz, J_1',2"_ = 5.9 Hz), 6.84 (d, 1H, NH, J_NH,CH-iPr_ = 7.6 Hz), 7.75 (s, 1H, H-2), 8.74 (s, 1H, CH=N).

^13^C NMR (75 MHz, DMSO-*d_6_*): δ = 22.9 & 23.3 (2 x CH_3_-iPr), 35.2 (2 x CH_3_-N), 38.2 (C-2'), 43.9 (CH-N-iPr), 61.4 (C-5'), 70.2 (C-3'), 79.6 (C-1'), 88.8 (C-4'), 125.1 (C-5), 148.9 (C-2), 149.2, 152.6 & 153.5 (C-4, C-6 & C-8), 159.9 (CH=N).

**8-*i*Butyl-amino-6-*N,N*-dimethylformamidine-2'-dA** (**10g)**

^1^H NMR (300 MHz, DMSO-*d_6_*): δ = 0.7-0.9 (m, 6H, 2 x CH_3_-iBut), 1.8-2.0 (m, 2H, H-2" & CH-iBut), 2.6-2.7 (m, 1H, H-2'), 2.95 (s, 3H, CH_3_-N), 3.1-3.3 (m, 5H, CH_2_-iBut & CH_3_-N), 3.6-3.7 (m, 2H, H-5' & 5"), 3.8-3.9 (m, 1H, H-4'), 4.4-4.5 (m, 1H, H-3'), 5.37 (d, 1H, OH-3', J_OH3',3'_ = 3.8 Hz), 5.65 (t, 1H, OH-5', J_OH5',5'_ = J_OH5',5"_ = 4.3 Hz), 6.31 (dd, 1H, H-1', J_1',2'_ = 9.0 Hz, J_1',2"_ = 5.6 Hz), 6.97 (t, 1H, NH, J_NH,CH2-iBut_ = 5.9 Hz), 7.98 (s, 1H, H-2), 8.90 (s, 1H, CH=N).

^13^C NMR (75 MHz, DMSO-*d_6_*): δ = 20.7 & 21.2 (2 x CH_3_-iBut), 27.9 (CH-iBut), 35.8 (2 x CH_3_-N), 38.7 (C-2'), 60.8 & 62.5 (C-5' & CH_2_-N-iBut), 71.7 (C-3'), 80.2 (C-1'), 90.5 (C-4'), 125.6 (C-5), 149.3 (C2), 153.2, 152.5 & 149.7 (C-4, C-6 & C-8), 160.1 (CH=N).

**6-*N,N*-Dimethylformamidine-8-methylthio-2'-dA** (**11)**

^1^H NMR (300 MHz, DMSO-*d_6_*): δ = 2.1-2.2 (m, 1H, H-2"), 2.65 (s, 3H, CH_3_-S), 3.07 (s, 3H, CH_3_-N), 3.0-3.2 (m, 4H, H-2' & CH_3_-N), 3.5-3.6 (2m, 2H, H-5' & H-5"), 3.8-3.9 (m, 1H, H-4'), 4.3-4.4 (m, 1H, H-3'), 5.3-5.4 (2m, 2H, OH-5' & OH-3'), 6.19 (t, 1H, H-1', J_1',2'_ = J_1',2"_ = 7.1 Hz), 7.91 (s, 1H, H-2), 8.79 (s, 1H, CH=N).

^13^C NMR (75 MHz, DMSO-*d_6_*): δ = 14.8 (CH_3_-S), 35.3 (2 x CH_3_-N), 36.9 (C-2'), 62.0 (C-5'), 71.3 (C-3'), 81.8 (C-1'), 89.2 (C-4'), 122.7 (C-5), 149.4 (C-2), 152.8 & 154.2 (C-4 & C-8), 155.5 (C-6), 158.9 (CH=N).

**8-Carbamoyl-6-*N,N*-dimethylformamidine-2'-dA** (**12)**

^1^H NMR (300 MHz, DMSO-*d_6_*): δ = 1.9-2.2 (2m, 2H, H-2' & H-2"), 3.12 & 3.24 (2s, 6H, 2 x CH_3_-N), 3.5-3.6 (m, 2H, H-5' & H-5"), 3.7-3.8 (m, 1H, H-4'), 4.1-4.2 (m, 1H, H-3'), 4.98 (t, 1H, OH-5', J_OH5',5'_ = J_OH5',5"_ = 5.8 Hz), 5.17 (d, 1H, OH-3', J_OH3',3'_ = 4.1 Hz), 6.03 (t, 1H, H-1', J_1',2'_ = J_1',2"_ = 6.9 Hz), 6.95 (s, 1H, NH_2_-amide), 7.27 (s, 1H, NH_2_-amide), 7.52 (s, 1H, H-2), 8.73 (s, 1H, CH=N).

^13^C NMR (75 MHz, DMSO- *d_6_*): δ = 35.4 (2 x CH_3_-N), 37.7 (C-2'), 61.9 (C-5'), 71.1 (C-3'), 83.7 (C-1'), 89.6 (C-4'), 121.8 (C-5), 150.1, 148.2, 152.0 & 153.7 (C-2, C-4, C-6 & C-8), 157.9 (CH=N), 162.8 (CO-amide).

**5'- *O*-Dimethoxytrityl-8-dimethylamino-6-*N,N*-dimethylformamidine-2'-dA (13a)**

^1^H NMR (300 MHz, DMSO-*d_6_*): δ = 2.0-2.1 (m, 1H, H-2"), 2.94 (s, 6H, 2 x CH_3_-N), 3.05 & 3.15 (2s, 6H, 2 x CH_3_-N), 3.3-3.5 (m, 3H, H-2', H-5' & H-5"), 3.56 (s, 6H, 2 x O-Me), 3.7-3.9 (m, 1H, H-4'), 4.3-4.4 (m, 1H, H-3'), 5.2 (br s, 1H, OH-3'), 6.08 (dd, 1H, H-1', J_1',2'_ = 6.3 Hz, J_1',2"_ = 5.8 Hz), 6.6-6.8 & 7.1-7.3 (2m, 13H, arom.), 7.95 (s, 1H, H-2), 8.80 (s, 1H, CH=N).

^13^C NMR (75 MHz, DMSO-*d_6_*): δ = 35.5 (2 x CH_3_-N), 38.7 (C-2'), 41.0 (2 x CH_3_-N), 54.7 (2 x OCH_3_), 61.9 (C-5'), 71.5 (C-3'), 83.9 (C-1'), 86.7 (C_IV_ DMTr), 88.7 (C-4'), 115.3 (4C arom.), 124.0 (C-5), 127.5, 129.7, 136.2 & 145.6 (12C arom.), 150.1, 150.8, 151.8 & 152.9 (C-2, C-4, C-6 & C-8), 158.1 & 158.2 (2C arom.), 159.4 (CH=N).

**5'- *O*-Dimethoxytrityl-6-*N,N*-dimethylformamidine-8-ethylmethylamino-2'-dA (13b)**

^1^H NMR (300 MHz, DMSO-*d_6_*): δ = 1.08 (t, 3H, CH_3_-CH_2_-N, J_CH3,CH2_ = 6.2 Hz), 2.1-2.2 (m, 1H, H-2"), 2.98 (s, 3H, CH_3_-N), 3.21 et 3.31 (2s, 6H, 2 x CH_3_-N), 3.34 (m, 1H, H-2'), 3.4-3.5 (m, 4H, H-5', H-5" & CH_2_-N), 3.6-3.7 (m, 1H, H-4'), 3.77 & 3.79 (2s, 6H, 2 x O-Me), 4.0-4.1 (m, 1H, H-3'), 4.98 (br s, 1H, OH-3'), 6.12 (t, 1H, H-1', J_1',2'_ = J_1',2"_ = 5.9 Hz), 6.7-6.8 & 7.1-7.4 (2m, 13H, arom.), 8.16 (s, 1H, H-2), 8.79 (s, 1H, CH=N).

^13^C NMR (75 MHz, DMSO-*d_6_*): δ = 12.5 (CH_3_-CH_2_-N), 35.7 & 36.2 (3 x CH_3_-N), 38.2 (C-2'), 48.7 (CH_2_-N), 54.6 (2 x OCH_3_), 66.2 (C-5'), 71.4 (C-3'), 83.1 (C-1'), 86.5 (C_IV_ DMTr), 88.5 (C-4'), 115.6 (4C arom.), 124.5 (C-5), 127.5, 128.5, 129.2, 136.0 & 144.9 (12C arom.), 149.2, 149.7, 152.5 & 153.1 (C-2, C-4, C-6 & C-8), 158.1 (2C arom.), 158.8 (CH=N).

**8-Diethylamino-5'-*O*-dimethoxytrityl-6-*N,N*-dimethylformamidine-2'-dA (13c)**

^1^H NMR (300 MHz, DMSO-*d_6_*): δ = 1.10 (t, 6H, 2 x CH_3_-CH_2_-N, J_CH3,CH2_ = 6.5 Hz), 2.0-2.2 (m, 1H, H-2"), 3.09 & 3.17 (2s, 6H, 2 x CH_3_-N), 3.2-3.3 (m, 7H, H-5', H-5", 2 x CH_2_-N & H-2'), 3.5-3.6 (m, 1H, H-4'), 3.66 & 3.68 (2s, 6H, 2 x O-Me), 4.0-4.2 (m, 1H, H-3'), 5.0 (br s, 1H, OH-3'), 6.11 (t, 1H, H-1', J_1',2'_ = J_1',2"_ = 6.2 Hz), 6.6-6.7 & 7.0-7.4 (2m, 13H, arom.), 8.22 (s, 1H, H-2), 8.84 (s, 1H, CH=N).

^13^C NMR (75 MHz, DMSO-*d_6_*): δ = 12.7 (CH_3_-CH_2_-N), 35.6 (2 x CH_3_-N), 38.7 (C-2'), 47.9 (2 x CH_2_-N), 55.1 (2 x OCH_3_), 65.8 (C-5'), 71.7 (C-3'), 82.4 (C-1'), 86.3 (C_IV_ DMTr), 87.9 (C-4'), 114.9 (4C arom.), 125.1 (C-5), 127.6, 128.3, 129.8, 136.3, 144.3 & 144.6 (12C arom.), 149.5, 149.8, 152.0 & 154.2 (C-2, C-4 C-6 & C-8), 158.2 & 158.3 (2C arom.), 158.6 (CH=N).

**5'-*O*-Dimethoxytrityl-6-*N,N*-dimethylformamidine-8-pyrrolidin-2'-dA (13d)**

^1^H NMR (300 MHz, DMSO-*d_6_*): δ = 1.7-1.9 (m, 4H, 2 x CH_2_-CH_2_-N), 2.1-2.3 (m, 1H, H-2"), 3.11 & 3.18 (2s, 6H, 2 x CH_3_-N), 3.2-3.4 (m, 7H, H-5', H-5", 2 x CH_2_-N & H-2'), 3.64 & 3.66 (2s, 6H, 2 x O-Me), 3.7-3.8 (m, 1H, H-4'), 4.2-4.4 (m, 1H, H-3'), 5.2 (br s, 1H, OH-3'), 6.05 (t, 1H, H-1', J_1',2'_ = J_1',2"_ = 7.2 Hz), 6.5-6.7 & 7.0-7.4 (2m, 13H, arom.), 8.10 (s, 1H, H-2), 8.74 (s, 1H, CH=N).

^13^C NMR (75 MHz, DMSO-*d_6_*): δ = 26.2 (2 x CH_2_-CH_2_-N), 35.3 (2 x CH_3_-N), 38.4 (C-2'), 48.9 (2 x CH_2_-N), 55.1 (2 x OCH_3_), 64.6 (C-5'), 71.9 (C-3'), 84.6 (C-1'), 86.1 (C_IV_ DMTr), 88.2 (C-4'), 114.6 (4C arom.), 124.1 (C-5), 127.3, 128.1, 129.4, 136.7 & 144.5 (12C arom.), 149.8, 150.2, 153.2 & 154.7 (C-2, C-4 C-6 & C-8), 157.5 & 157.6 (2C arom.), 158.2 (CH=N).

**5'-*O*-Dimethoxytrityl-6-*N,N*-dimethylformamidine-8-piperidin-2'-dA (13e)**

^1^H NMR (300 MHz, DMSO-*d_6_*): δ = 1.6-1.9 (m, 6H, 3 x CH_2_- *pip*), 2.1-2.2 (m, 1H, H-2"), 3.11 & 3.15 (2s, 6H, 2 x CH_3_-N), 3.2-3.5 (m, 7H, H-5', H-5", 2 x CH_2_-N & H-2'), 3.63 & 3.65 (2s, 6H, 2 x O-Me), 3.7-3.8 (m, 1H, H-4'), 4.3-4.5 (m, 1H, H-3'), 5.3 (br s, 1H, OH-3'), 6.01 (t, 1H, H-1', J_1',2'_ = J_1',2"_ = 6.5 Hz), 6.5-6.8 & 7.1-7.4 (2m, 13H, arom.), 8.08 (s, 1H, H-2), 8.82 (s, 1H, CH=N).

^13^C NMR (75 MHz, DMSO-*d_6_*): δ = 24.5 (CH_2_-Nγ-pip), 25.3 (2 x CH_2_-Nβ-pip), 35.1 (2 x CH_3_-N), 38.6 (C-2'), 49.1 (2 x CH_2_-N), 55.3 (2 x OCH_3_), 64.3 (C-5'), 72.1 (C-3'), 84.9 (C-1'), 86.3 (C_IV_ DMTr), 88.1 (C-4'), 114.4 (4C arom.), 123.8 (C-5), 127.1, 128.4, 129.2, 136.5 & 144.7 (12C arom.), 149.5, 151.1, 153.9 & 155.1 (C-2, C-4 C-6 & C-8), 157.7 & 157.8 (2C arom.), 158.0 (CH=N).

**8-*i*Propyl-amino-5'-*O*-dimethoxytrityl-6-*N,N*-dimethylformamidine-2'-dA (13f)**

^1^H NMR (300 MHz, DMSO-*d_6_*): δ = 1.24 & 1.26 (2s, 6H, 2 x CH_3_-iPr), 2.0-2,1 (m, 1H, H-2"), 2.5-2.6 (m, 2H, H-2' & CH-iPr), 3.04 & 3.16 (2s, 6H, 2 x CH_3_-N), 3.0-3.2 (m, 2H, H-5' & 5"), 3.69 & 3.71 (2s, 6H, 2 x O-Me), 3.9-4.0 (m, 1H, H-4'), 4.4-4.5 (m, 1H, H-3'), 5.28 (d, 1H, OH-3', J_OH3',3'_ = 6.1 Hz), 6.1-6.2 (m, 2H, H-1' & NH-iPr), 6.7-6.8 & 7.1-7.3 (2m, 13H, arom.), 7.91 (s, 1H, H-2), 8.82 (s, 1H, CH=N).

^13^C NMR (75 MHz, DMSO-*d_6_*): δ = 22.8 & 23.1 (2 x CH_3_-iPr), 35.6 (2 x CH_3_-N), 38.5 (C-2'), 42.8 (CH-N-iPr), 54.4 (2 x OCH_3_), 65.7 (C-5'), 71.1 (C-3'), 82.6 (C-1'), 86.6 (C_IV_ DMTr), 88.6 (C-4'), 114.8 (4C arom.), 125.3 (C-5), 127.9, 128.5, 129.4, 136.5 & 144.8 (12C arom.), 149.1, 149.9, 152,7 & 153,4 (C-2, C-4, C-6 & C-8), 158.0 (2C arom.), 159.3 (CH=N).

**8-*i*Butyl-amino-5'-*O*-dimethoxytrityl-6-*N,N*-dimethylformamidine-2'-dA (13g)**

^1^H NMR (300 MHz, DMSO-*d_6_*): δ = 0.8-0.9 (m, 6H, 2 x CH_3_-iBut), 1.9-2.1 (2m, 2H, H-2" & CH-iBut), 2.6-2.7 (m, 1H, H-2'), 2.98 (s, 3H, CH_3_-N), 3.0-3.1 (m, 6H, H-5", CH_2_-iBu & CH_3_-N), 3.2-3.3 (m, 1H, H-5'), 3.70 (s, 6H, 2 x O-Me), 3.9-4.0 (m, 1H, H-4'), 4.5-4.6 (m, 1H, H-3'), 5.29 (d, 1H, OH-3', J_OH3',3'_ = 6.0 Hz), 6.2-6.3 (m, 2H, NH-iBut & H-1'), 6.7-6.8 & 7.1-7.3 (2m, 13H, arom.), 7.98 (s, 1H, H-2), 8.90 (s, 1H, CH=N).

^13^C NMR (75 MHz, DMSO-*d_6_*): δ = 18.5 & 19.2 (2 x CH_3_-iBut), 27.2 (CH-iBut), 35.7 (2 x CH_3_-N), 38.9 (C-2'), 54.7 (2 x OCH_3_), 61.1 (CH_2_-N-iBut), 66.8 (C-5'), 71.4 (C-3'), 81.4 (C-1'), 88.0 (C_IV_ DMTr), 89.8 (C-4'), 115.2 (4C arom.), 125.7 (C-5), 127.6, 128.1, 129.2, 134.8, 144.5 & 144.7 (12C arom.), 149.6, 150.1, 152.8 & 153.3 (C-2, C-4, C-6 & C-8), 158.2 (2C arom.), 160.0 (CH=N).

**5'-*O*-Dimethoxytrityl-6-*N,N*-dimethylformamidine-8-methylthio-2'-dA (14)**

^1^H NMR (300 MHz, DMSO-*d_6_*): δ = 2.1-2.2 (m, 1H, H-2"), 2.69 (s, 3H, CH_3_-S), 3.0-3.2 (m, 8H, H-5', H-5" & 2 x CH_3_-N), 3.3 (m, 1H, H-2'), 3.66 & 3.68 (2s, 6H, 2 x O-Me), 3.9-4.0 (m, 1H, H-4'), 4.5-4.6 (m, 1H, H-3'), 5.33 (d, 1H, OH-3', J_OH3',3'_ = 6.0 Hz), 6.20 (t, 1H, H-1', J_1',2'_ = J_1',2"_ = 7.5 Hz), 6.6-6.8 & 7.1-7.3 (2m, 13H, arom.), 8.16 (s, 1H, H-2), 8.81 (s, 1H, CH=N).

^13^C NMR (75 MHz, DMSO-*d_6_*): δ = 14.6 (CH_3_-S), 36.11 (2 x CH_3_-N), 38.7 (C-2'), 54.8 (2 x OCH_3_), 62.5 (C-5'), 71.0 (C-3'), 80.9 (C-1'), 87.3 (C_IV_ DMTr), 88.7 (C-4'), 115.3 (4C arom.), 125.7 (C-5), 127.0, 128.5, 130.3, 136.4 & 145.8 (12C arom.), 146.7, 152.5, 152.8 & 153.4 (C-2, C-4, C-6 & C-8), 158.0 & 158.3 (2C arom.), 159.4 (CH=N).

**8-Carbamoyl-5'-*O*-dimethoxytrityl-6-*N,N*-dimethylformamidine-2'-dA (15)**

^1^H NMR (300 MHz, DMSO-*d_6_*): δ = 1.9-2.1 & 2.8-2.9 (2m, 2H, H-2' & H-2"), 2.9-3.1 (m, 5H, H-5', H-5" & CH_3_-N), 3.20 (s, 3H, CH_3_-N), 3.58 (s, 6H, 2 x O-Me), 3.7-3.8 (m, 1H, H-4'), 4.3-4.4 (m, 1H, H-3'), 5.19 (d, 1H, OH-3', J_OH3',3'_ = 5.3 Hz), 6.14 (t, 1H, H-1', J_1',2'_ = J_1',2"_ = 6.8 Hz), 6.6-6.8 & 7.1-7.3 (2m, 15H, arom. & NH_2_-amide), 7.85 (s, 1H, H-2), 8.76 (s, 1H, CH=N).

^13^C NMR (75 MHz, DMSO- *d_6_*): δ = 37.1 (2 x CH_3_-N), 39.0 (C-2'), 54.9 (2 x OCH_3_), 62.2 (C-5'), 71.3 (C-3'), 84.0 (C-1'), 87.1 (C_IV_ DMTr), 88.5 (C-4'), 115.2 (4C arom.), 123.3 (C-5), 127.2, 128.4, 130.2, 136.4 & 145.7 (12C arom.), 148.4, 149.3, 152.2 & 153.6 (C-2, C-4, C-6 & C-8), 158.0, 158.3 & 158.4 (CH=N & 2C arom.), 162.6 (CO-amide).

**5'-*O*-Dimethoxytrityl-8-ethyl-2'-dA (18)**

ESI MS (+) (m/z): Calculated for C_33_H_35_N_5_O_5_H [M+H]^+^: 582.3; found: 581.7.

^1^H NMR (200 MHz, CDCl_3_): δ = 1.2-1.3 (t, 3H, CH_3_, J_CH3,CH2_ = 7.5 Hz,), 2.1-2.2 (m, 1H, H2’), 2.8-2.9 (q, 2H, CH_2_, J_CH3,CH2_ = 7.5 Hz,), 3.2-3.3 (m, 2H, H5’), 3.4-3.5 (m, 1H, H2’), 3.65 (s, 6H, CH_3_O), 3.9-4.0 (m, 1H, H4’), 4.8-4.9 (m, 1H, H3’), 5.73 (s, 2H, NH_2_), 6.2 (t, 1H, H1’, J_H1’,H2’_ =J_H1’,H2”_ = 6.82 Hz,), 6.6-6.7 (m, 4H, arom.), 7.0-7.3 (m, 9H, arom.), 7.97 (s, 1H, H2),

^13^C NMR (75 MHz, CDCl_3_): δ =11.8 (CH_3_), 21.7 (CH_2_), 37.1 (C2’), 55.1 (2 x CH_3_O), 63.7 (C5’), 72.6 (C4’), 84.0 (C1’), 85.7 (C3’), 112.9 (4C arom.), 126-129 (10C arom. & C5), 135.9 & 144.7 (2C arom.), 150.7, 151.7, 154.3 & 154.6 (C2, C4, C6, C8), 158.3 (2C arom.).

**5'-*O*-Dimethoxytrityl-6-*N,N*-dimethylformamidine-8-ethyl-2'-dA (19)**

ESI MS (+) (m/z): Calculated for C_36_H_40_N_6_O_5_H [M+H]^+^: 637.3; found: 636.9.

^1^H NMR (200 MHz, CDCl_3_): δ = 1.63 (t, 3H, CH_3_, J_CH3,CH2_ = 7.5 Hz,), 2.3-2.4 (m, 1H, H2’), 2.98 (q, 2H, CH_2_, J_CH3,CH2_ = 7.5 Hz,), 3.17 & 3.24 (2s, 6H, 2 x CH_3_N), 3.4-3.5 (m, 2H, H5’ & H5”), 3.5-3.6 (m, 1H, H2’), 3.70 (6H, s, 2 x CH_3_O), 4.0-4.1 (m, 1H, H4’), 4.9-5.0 (m, 1H, H3’), 6.30 (t, 1H, H1’, J_H1’,H2’_ =J_H1’,H2”_ = 6.87 Hz), 6.7-6.8 & 7.2- 7.4 (m, 13H, arom.), 8.27 (s, 1H, H2), 8.84 (s, 1H, CH=N).

^13^C NMR (75 MHz, CDCl_3_): δ = 12.1 (CH_3_), 21.8 (CH_2_), 35.1 (N-CH_3_), 37.2 (C2’), 41.2 (N-CH_3_), 55.2 (2 x OCH_3_), 63.8 (C5’), 73.1 (C4’), 83.8 (C3’), 85.5 (C1’), 113 (4C arom.), 126-145 (12C arom. & C5), 148.2, 149.6, 151.3, 152.7 (C2, C4, C6 & C8), 157.7, 158.4 & 158.9 (CH=N & 2C arom.).

**3'-*N,N*-Diisopropylcyanoethylphosphoramidite-5'-*O*-dimethoxytrityl-8-dimethylamino-6-*N,N*-dimethylformamidine-2'-dA (20a)**

^1^H NMR (300 MHz, DMSO-*d_6_*): δ = 1.0-1.3 (m, 12H, CH_3_-iPr), 2.4-2.5 (m, 1H, H-2"), 2.6-2.7 (m, 2H, CH_2_-CN), 2.97, 3.15 & 3,25 (3s, 12H, 4 x CH_3_-N), 3.3-3.5 (2m, 7H, H-2', H-5', H-5", 2 x CH-iPr & CH_2_O), 3.78 (s, 6H, 2 x O-Me), 4.4-4.5 (m, 1H, H-4'), 5.0-5.1 (m, 1H, H-3'), 6.23 (t, 1H, H-1', J_1',2'_ = J_1',2"_ = 6.2 Hz), 6.7-6.8 & 7.2-7.4 (2m, 13H, arom.), 8.01 (s, 1H, H-2), 8.71 (s, 1H, CH=N).

**3'-*N,N*-Diisopropylcyanoethylphosphoramidite-5'-*O*-dimethoxytrityl-6-*N,N*-dimethylformamidine-8-ethylmethylamino-2'-dA (20b)**

^1^H NMR (300 MHz, DMSO-*d_6_*): δ = 1.1-1.4 (m, 15H, CH_3_-CH_2_-N & CH_3_-iPr), 2.5-2.6 (m, 1H, H-2"), 2.6-2.7 (m, 2H, CH_2_-CN), 3.01, 3.15 & 3.21 (3s, 9H, 3 x CH_3_-N), 3.3-3.7 (2m, 9H, H-2', H-5', H-5", 2 x CH-iPr, CH_2_N & CH_2_O), 3.75 (s, 6H, 2 x O-Me), 4.4-4.6 (m, 1H, H-4'), 5.0-5.1 (m, 1H, H-3'), 6.12 (t, 1H, H-1', J_1',2'_ = J_1',2"_ = 6.7 Hz), 6.6-6.8 & 7.1-7.4 (2m, 13H, arom.), 7.96 (s, 1H, H-2), 8.13 (s, 1H, CH=N).

**3'-*N,N*-Diisopropylcyanoethylphosphoramidite-5'-*O*-dimethoxytrityl-6-*N,N*-dimethylformamidine-8-diethylamino-2'-dA (20c)**

^1^H NMR (300 MHz, DMSO-*d_6_*): δ = 1.1-1.4 (m, 18H, 2 x CH_3_-CH_2_-N & CH_3_-iPr), 2.3-2.4 (m, 1H, H-2"), 2.7-2.8 (m, 2H, CH_2_-CN), 3.17 (s, 3H, CH_3_-N), 3.2-3.8 (2m, 14H, H-2', H-5', H-5", 2 x CH-iPr, 2 x CH_2_-N, CH_2_O & CH_3_N), 3.77 (s, 6H, 2 x O-Me), 4.4-4.5 (m, 1H, H-4'), 4.9-5.0 (m, 1H, H-3'), 6.15 (br s, 1H, H-1'), 6.6-6.8 & 7.1-7.4 (2m, 13H, arom.), 8.02 (s, 1H, H-2), 8.24 (s, 1H, CH=N).

**3'-N,N-Diisopropylcyanoéthylphosphoramidite-5'-diméthoxytrityl-6-N,N-diméthylformamidine-8-pyrrolidin-2'-dA (20d)**

^1^H NMR (300 MHz, DMSO-*d_6_*): δ = 1.3-1.8 (m, 16H, 2 x CH_2_-CH_2_-N & 3 x CH_3_-iPr), 2.2-2.3 (m, 1H, H-2"), 2.7-2.8 (m, 2H, CH_2_-CN), 3.15 (s, 3H, CH_3_-N), 3.2-3.7 (2m, 14H, H-2', H-5', H-5", 2 x CH-iPr, 2 x CH_2_-N, CH_2_-O & CH_3_-N), 3.71 (s, 6H, 2 x O-Me), 4.1-4.2 (m, 1H, H-4'), 4.8-4.9 (m, 1H, H-3'), 6.1 (br s, 1H, H-1'), 6.6-6.8 & 7.0-7.4 (2m, 13H, arom.), 8.06 (s, 1H, H-2), 8.62 (s, 1H, CH=N).

**3'-N,N-Diisopropylcyanoéthylphosphoramidite-5'-diméthoxytrityl-6-N,N-diméthylformamidine-8-piperidin-2'-dA (20e)**

^1^H NMR (300 MHz, DMSO-*d_6_*): δ = 1.3-1.9 (m, 18H, 3 x CH_2_-*pip* & 3 x CH_3_-iPr), 2.1-2.2 (m, 1H, H-2"), 2.6-2.8 (m, 2H, CH_2_-CN), 3.18 (s, 3H, CH_3_-N), 3.2-3.7 (2m, 14H, H-2', H-5', H-5", 2 x CH-iPr, 2 x CH_2_-N, CH_2_-O & CH_3_-N), 3.75 (s, 6H, 2 x O-Me), 3.9-4.0 (m, 1H, H-4'), 4.9-5.0 (m, 1H, H-3'), 6.0 (br s, 1H, H-1'), 6.5-6.9 & 7.1-7.4 (2m, 13H, arom.), 8.04 (s, 1H, H-2), 8.75 (s, 1H, CH=N).

**3'-*N,N*-Diisopropylcyanoethylphosphoramidite-5'-*O*-dimethoxytrityl-6-*N,N*-dimethylformamidine-8-isopropyl-amino-2'-dA (20f)**

^1^H NMR (300 MHz, DMSO-*d_6_*): δ = 1.0-1.3 (2m, 18H, 6 x CH_3_-iPr), 2.3-2.4 (m, 1H, H-2"), 2.5-2.7 (m, 2H, CH_2_-CN), 3.11 & 3.19 (2s, 6H, 2 x CH_3_-N), 3.2-3.6 (2m, 8H, H-2', H-5', 5", 3 x CH-iPr & CH_2_-O), 3.76 (s, 6H, 2 x O-Me), 4.0-4.1 (m, 1H, H-4'), 4.6-4.7 (m, 1H, H-3'), 6.15 (dd, 1H, H-1', J_1',2'_ = 8.9 Hz, J_1',2"_ = 5.8 Hz), 6.44 (sl, 1H, NH-iPr), 6.7-6.8 & 7.2-7.4 (2m, 13H, arom.), 7.76 (s, 1H, H-2), 8.02 (s, 1H, CH=N).

**3'-*N,N*-Diisopropylcyanoethylphosphoramidite-5'-*O*-dimethoxytrityl-6-*N,N*-dimethylformamidine-8-isobutyl-amino-2'-dA (20g)**

^1^H NMR (300 MHz, DMSO-*d_6_*): δ = 1.1-1.3 (2m, 18H, CH_3_-iBut & CH_3_-iPr), 2.2-2.7 (2m, 4H, H-2", CH-iBu & CH_2_-CN), 3.0-3.1 (m, 5H, CH_2_-iBu & CH_3_-N), 3.24 (s, 3H, CH_3_-N), 3.3-3.4 (m, 1H, H-2'), 3.4-3.7 (m, 6H, H-5', 5", 2 x CH-iPr & CH_2_-O), 3.75 (s, 6H, 2 x O-Me), 4.2-4.3 (m, 1H, H-4'), 4.7-4.8 (m, 1H, H-3'), 6.47 (t, 1H, H-1', J_1',2'_ = J_1',2"_ = 7.5 Hz), 6.7-6.8 & 7.1-7.4 (2m, 13H, arom.), 8.07 (t, 1H, NH, J_NH,CH2-iBut_ = 6.4 Hz), 8.48 (s, 1H, H-2), 8.92 (s, 1H, CH=N).

**3'-*N,N*-Diisopropylcyanoethylphosphoramidite-5'-*O*-dimethoxytrityl-6-*N,N*-dimethylformamidine-8-methylthio-2'-dA (21)**

^1^H NMR (300 MHz, DMSO-*d_6_*): δ = 1.1-1.3 (m, 12H, CH_3_-iPr), 2.3-2.5 (m, 2H, CH_2_-CN), 2.6-2.7 (m, 1H, H-2"), 2.78 (s, 3H, CH_3_-S), 3.17 & 3.24 (2s, 6H, CH_3_-N), 3.3-3.4 (m, 1H, H-2'), 3.4-3.7 (m, 6H, H-5', H-5", 2 x CH-iPr & CH_2_O), 3.76 (s, 6H, 2 x O-Me), 4.2-4.3 (m, 1H, H-4'), 4.8-5.0 (m, 1H, H-3'), 6.35 (t, 1H, H-1', J_1',2'_ = J_1',2"_ = 6.4 Hz), 6.7-6.8 & 7.1-7.4 (2m, 13H, arom.), 8.24 (s, 1H, H-2), 8.88 (s, 1H, CH=N).

**8-Carbamoyl-3'-*N,N*-diisopropylcyanoethylphosphoramidite-5'-*O*-dimethoxytrityl-6-*N,N*-dimethylformamidine-2'-dA (22)**

^1^H NMR (300 MHz, DMSO-*d_6_*): δ = 1.1-1.3 (m, 12H, CH_3_-iPr), 2.4-2.5 (m, 2H, CH_2_-CN), 2.5-2.6 (m, 1H, H-2"), 3.01 & 3,09 (2s, 6H, CH_3_-N), 3.3-3.6 (2m, 7H, H-2', H-5', H-5", CH_2_O & 2 x CH-iPr), 3.79 (s, 6H, 2 x O-Me), 4.1-4.2 (m, 1H, H-4'), 4.7-4.9 (m, 1H, H-3'), 6.14 (m, 1H, H-1'), 6.8-6.9 & 7.2-7.4 (2m, 13H, arom.), 7.74 (s, 1H, H-2), 8.85 (s, 1H, CH=N).

**3'-*N,N*-Diisopropylcyanoethylphosphoramidite-5'-*O*-dimethoxytrityl-6-*N,N-*dimethylformamidine-8-ethyl-2'-dA (24)**

^1^H NMR (200 MHz, CD_2_Cl_2_): δ = 1.1-1.3 (m, 12H, CH_3_), 1.47 (t, 3H, CH_3_, J_CH3,CH2_ = 7.5 Hz,), 2.4-2.5 (m, 1H, H2’), 2.5-2.6 (m, 1H, CH), 2.6-2.7 (m, 1H, CH), 3.05 (q, 2H, CH_2_, J_CH3,CH2_ = 7.3 Hz,), 3.21 & 3.25 (2s, 6H, 2 x CH_3_N), 3.4-3.5 (m, 2H, CH_2_CN), 3.6-3.8 (m, 5H, CH_2_O, H2’ & H5’), 3.80 (s, 6H, 2 x CH_3_O), 4.2-4.3 (m, 1H, H4’), 5.0-5.1 (m, 1H, H3’), 6.37 (dd, 1H, H1’, J_H1’,H2’_ = 4.3 Hz & J_H1’,H2”_ = 6.7 Hz), 6.8-6.9 & 7.2-7.4 (m, 13H, arom.), 8.27 (s, 1H, H2), 8.91 (s, 1H, CH=N).
