# Supplementary material for: 8-Modified-2′-Deoxyadenosine Analogues Induce Delayed Polymerization Arrest during HIV-1 Reverse Transcription
Source: PLoS One. 2011 Nov 7;6(11):e27456. doi: 10.1371/journal.pone.0027456 (PMC3210175; doi:10.1371/journal.pone.0027456)
Supplement: Table S1 — Analysis of compounds 10a–g, 11, 12, 13a–g, 14 and 15. Starting quantities, yields and mass analyses. (DOCX) [file pone.0027456.s005.docx]

|  | Starting material | Yield | Molecular formula | Mass Spectrometry | |
| --- | --- | --- | --- | --- | --- |
|  | mg / mmol | % |  | Calcd | Found |
| **10 a** | 350 / 1.19 | 89 | C_15_H_23_N_7_O_3_Na (M+Na)^+^ | 372,1760 | 372,1896 |
| **10 b** | 400 / 1.30 | 78 | C_16_H_25_N_7_O_3_H (M+H)^+^ | 364,2097 | 364,2098 |
| **10 c** | 385 / 1.20 | 82 | C_17_H_27_N_7_O_3_Na (M+Na)^+^ | 400,2073 | 400,2354 |
| **10 d** | 410 / 1.28 | 81 | C_17_H_25_N_7_O_3_H (M+H)^+^ | 376.2097 | 376.2206 |
| **10 e** | 400 / 1.20 | 79 | C_18_H_27_N_7_O_3_H (M+H)^+^ | 390.2254 | 390.3021 |
| **10 f** | 240 / 0.78 | 75 | C_16_H_25_N_7_O_3_Na (M+Na)^+^ | 386,1917 | 386,2256 |
| **10 g** | 270 / 0.84 | 79 | C_17_H_27_N_7_O_3_H (M+H)^+^ | 378,2254 | 378,2386 |
| **11** | 360 / 1.21 | 86 | C_14_H_20_N_6_O_3_SNa [M+Na]^+^ | 375.1215 | 375.1905 |
| **12** | 235 / 0.80 | 71 | C_14_H_19_N_7_O_4_Na [M+Na]^+^ | 372.1396 | 372.1518 |
| **13a** | 360 / 1.03 | 68 | C_36_H_41_N_7_O_5_Na (M+Na)^+^ | 674.3067 | 674.3197 |
| **13b** | 355 / 0.98 | 67 | C_37_H_43_N_7_O_5_Na (M+Na)^+^ | 688.3223 | 688.3106 |
| **13c** | 362 / 0.96 | 75 | C_38_H_45_N_7_O_5_Na (M+Na)^+^ | 702.3380 | 702.3352 |
| **13d** | 350 / 0.93 | 71 | C_38_H_43_N_7_O_5_Na (M+Na)^+^ | 700.3223 | 700.3156 |
| **13e** | 310 / 0.80 | 69 | C_39_H_45_N_7_O_5_Na (M+Na)^+^ | 714.3380 | 714.3235 |
| **13f** | 205 / 0.56 | 71 | C_37_H_43_N_7_O_5_Na (M+Na)^+^ | 688.3223 | 688.3274 |
| **13g** | 240 / 0.64 | 75 | C_38_H_45_N_7_O_5_H (M+H)^+^ | 680.3560 | 680.3429 |
| **14** | 355 / 1.01 | 69 | C_35_H_38_N_6_O_5_SNa [M+Na]^+^ | 677.2522 | 677.2455 |
| **15** | 190 / 0.54 | 72 | C_35_H_37_N_7_O_6_Na [M+Na]^+^ | 674.2703 | 674.2810 |
