# Supplementary material for: 8-Modified-2′-Deoxyadenosine Analogues Induce Delayed Polymerization Arrest during HIV-1 Reverse Transcription
Source: PLoS One. 2011 Nov 7;6(11):e27456. doi: 10.1371/journal.pone.0027456 (PMC3210175; doi:10.1371/journal.pone.0027456)
Supplement: Table S2 — Analysis of compounds 20a–g, 21, 22, 23 and 24. Starting quantities, yields, mass analyses and 31P NMR. (DOCX) [file pone.0027456.s006.docx]

|  | Starting material | Yield | MS | ^31^P NMR |
| --- | --- | --- | --- | --- |
|  | mg / mmol | % |  |  |
| **20a** | 449 / 0.69 | 86 | 874.6 (M+Na)^+^ | 150.26 / 149.83 |
| **20b** | 430 / 0.65 | 81 | 888.7 (M+Na)^+^ | 149.84 / 149.54 |
| **20c** | 482 / 0.71 | 90 | 902.9 (M+Na)^+^ | 150.36 / 150.14 |
| **20d** | 440 / 0.65 | 89 | 900.6 (M+Na)^+^ | 150.32 / 150.08 |
| **20e** | 363 / 0.53 | 86 | 914.5 (M+Na)^+^ | 150.16 / 149.78 |
| **20f** | 255 / 0.39 | 87 | 888.9 (M+Na)^+^ | 150.24 / 149.95 |
| **20g** | 318 / 0.47 | 85 | 902.7 (M+Na)^+^ | 150.05 / 149.86 |
| **21** | 449 / 0.66 | 88 | 855.5 (M+H)^+^ | 150.01 / 149.64 |
| **22** | 247 / 0.38 | 80 | 874.8 (M+Na)^+^ | 150.41 / 150.15 |
| **23** | 300 / 0.47 | 60 | 835.2 (M+H)^+^ | 149.9 / 149.2 |
| **24** | 313 / 0.49 | 70 | 859.8 (M+Na)^+^ | 149.1 / 148.9 |
