# Supplementary material for: 8-Modified-2′-Deoxyadenosine Analogues Induce Delayed Polymerization Arrest during HIV-1 Reverse Transcription
Source: PLoS One. 2011 Nov 7;6(11):e27456. doi: 10.1371/journal.pone.0027456 (PMC3210175; doi:10.1371/journal.pone.0027456)
Supplement: Table S3 — Retention time on anion exchange HPLC column and Maldi-TOF mass of oligodeoxynucleotides. HPLC was performed on a Dionex DNA-PacTM 28 PA-100 anion exchange column (9×250 mm) at 60°C with the following gradient system (A = 4 M urea 0.2% acetonitrile − 20 mM Mes buffer pH 6.5 − 1 mM NaClO4 and B = 4 M urea − 0.2% acetonitrile − 20 mM Mes buffer pH 6.5 − 400 mM NaClO4) from 15 to 70% of solution B in solution A in 50 min at a 1 ml/min flow rate. (DOC) [file pone.0027456.s007.doc]

|  | Lenght | Tr HPLC min | SM – Maldi-TOF (M-H)^-^ | |
| --- | --- | --- | --- | --- |
| Modification X |  |  | Calculated | Found |
| **8-Me_2_N-2'-dA** | 18 mer | 28.74 | 5509.769 | 5510.643 |
|  | 19 mer | 29.48 | 5798.673 | 5799.481 |
|  | 31 mer | 33.97 | 9581.482 | 9584.649 |
| **8-Et,Me-N-2'-dA** | 18 mer | 29.23 | 5523.683 | 5524.386 |
|  | 19 mer | 30.82 | 5812.868 | 5813.773 |
|  | 31 mer | 34.08 | 9595.362 | 9585.172 |
| **8-Et_2_-N-2'-dA** | 18 mer | 29.38 | 5537.937 | 5538.638 |
|  | 19 mer | 30.05 | 5826.264 | 5827.593 |
|  | 31 mer | 34.17 | 9609.398 | 9611.418 |
| **8-iPr-N-2'-dA** | 18 mer | 30.41 | 5523.683 | 5524.699 |
|  | 19 mer | 31.12 | 5812.868 | 5813.448 |
|  | 31 mer | 33.48 | 9595.362 | 9596.378 |
| **8-iBu-N-2'-dA** | 18 mer | 30.29 | 5537.715 | 5538.233 |
|  | 19 mer | 30.68 | 5826.895 | 5827.821 |
|  | 31 mer | 34.25 | 9610.389 | 9611.898 |
| **8-MeS-2'-dA** | 18 mer | 30.22 | 5512.577 | 5512.988 |
|  | 19 mer | 30.49 | 5801.762 | 5803.289 |
|  | 31 mer | 34.43 | 9584.256 | 9583.450 |
| **8-Et-2’-dA** | 18 mer | 19.48 | 5494.59 | 5491.92 |
|  | 19 mer | 19.54 | 5783.76 | 5778.38 |
|  | 31 mer | 22.20 | 9566.267 | 9560.921 |
